# Multiomic analysis of papillary thyroid cancers identifies BAIAP2L1-BRAF fusion and requirement of TRIM25, PDE5A and PKCδ for tumorigenesis

**DOI:** 10.1186/s12943-022-01665-y

**Published:** 2022-10-10

**Authors:** Emilie Renaud, Kristina Riegel, Rossana Romero, Kushal Suryamohan, Ute Distler, Stefan Tenzer, Arno Schad, Thomas J. Musholt, Krishnaraj Rajalingam

**Affiliations:** 1 Cell Biology Unit, University Medical Center of the Johannes Gutenberg University Mainz, 55131 Mainz, Germany; 2 MedGenome Inc, Foster City, CA 94404 USA; 3 Institute of Immunology, University Medical Center of the Johannes Gutenberg University Mainz, 55131 Mainz, Germany; 4 Institute of Pathology, University Medical Center of the Johannes Gutenberg University Mainz, 55131 Mainz, Germany; 5 Endocrine Surgery Section, Department of General, Visceral and Transplantation Surgery, University Medicine, 55131 Mainz, Germany

**Keywords:** Papillary thyroid cancer, PTC, BAIAP2L1-BRAF, TRIM25, PKCδ, Precision medicine, Proteomics, Interactome

## Abstract

**Background:**

Papillary thyroid carcinoma (PTC) is one of the most common forms of thyroid cancer with a cure rate of over 90% after surgery. However, aggressive forms may still occur, and personalized therapeutic strategies are increasingly required.

**Methods:**

We performed integrated genomic and proteomic analysis of PTC tumor samples from patients who did not harbor BRAF or RAS mutations. We validate the analysis and present in-depth molecular analysis of the identified genetic rearrangement by employing biochemical and cell biological assays. Finally, we employ 3D spheroid models, loss of function studies and chemical inhibitors to target the hitherto upregulated factors. The data are analysed with appropriate statistical tests which are mentioned in the legends section.

**Results:**

In a 23-year-old patient with thyroiditis, we identified a novel rearrangement leading to a BAIAP2L1-BRAF fusion that transforms immortalized human thyroid cells in a kinase and CC-domain dependent manner. Moreover, quantitative proteomic analysis of the same patient samples revealed the upregulation of several proteins including the Ubiquitin E3 ligase TRIM25, PDE5A, and PKCδ. Further, in a cohort of PTC patients, we observed higher expression of TRIM25 and PKCδ in the tumor and metastatic lesions, when compared to the matched normal tissue. Inhibition of TRIM25, PDE5A and PKCδ with small molecules or RNA interference affected not only viability and proliferation of BAIAP2L1-BRAF transformed cells, but also the viability, growth and invasion of corresponding 3D spheroid cultures.

**Conclusions:**

Apart from unveiling a novel oncogenic BRAF fusion in PTCs, our data may open a novel avenue of therapeutic targeting in human PTCs.

**Supplementary Information:**

The online version contains supplementary material available at 10.1186/s12943-022-01665-y.

## Background

The incidence of thyroid cancer has steadily increased over the past three decades and may become the fourth most frequent cancer by 2030. Papillary thyroid carcinoma (PTC), the most common form of thyroid cancer, accounts for 80% of all thyroid cancer cases [1]. The development of lymph node (LN) metastases is usually the most common risk of recurrence (1.7–15% of patients). Distant metastases are rare in PTC, but the presence of metastases is associated with poor prognosis [2]. Apart from that, prognosis is directly related to tumor size, age, and gender, with females being more commonly affected than males by a ratio of 3:1 [3]. Although overall survival from PTC is good, refractory aggressive forms remain a major therapeutic challenge. In a recent study of 148 pediatric cases [4], 5% of patients died from the disease at a mean age of 30.7 years. Currently, there is neither a predictive method nor an alternative treatment regimen for advanced PTC. Improved diagnosis and prognosis of PTC depend on a better understanding of the genetic basis and biology of the disease.

The most frequent driver mutations in PTC are BRAF mutation V600E and RAS mutations. Moreover, PTC can be caused by genetic rearrangements that are usually mutually exclusive with BRAF and RAS mutations. Gene fusions involving kinases account for the majority of PTC-associated genetic rearrangements. RET kinase rearrangements occur in approximately 14% of PTC [5]. Other gene fusions involve kinases like AGK, BRAF, NTRK or ALK. According to Lan et al. [5], gene fusions and copy number variations (CNVs) rather than V600E mutations are linked to an increased risk of developing LN metastases. For example, the AGK-BRAF fusion is a common event in pediatric PTC that promotes an aggressive phenotype and is associated with higher levels of telomere-related genomic instability.

Fusion genes involving oncogenic kinases are attractive therapeutic targets in precision medicine since they can be easily targeted by inhibitors. Therefore, identifying and characterizing new gene fusions involved in the development of PTC is a promising approach to improve the stratification of thyroid lesions, preoperative diagnosis and to provide new personalized treatments. An integrative analysis of genomic, transcriptomic, as well as proteomic characteristics of PTCs will also help to identify relevant targetable alterations in PTC. Here, by performing RNA-seq analysis, we identified a novel BRAF fusion (BAIAP2L1-BRAF). Proteomic analysis also identified several druggable targets. In particular, we found that TRIM25 and PKCδ are critically required for BRAF-driven PTCs.

## Methods

### Patient sample acquisition

Informed consent was obtained from patients before surgery. Ethical approval from the State Medical Board, which is also the institutional approval (study number: 9888), was obtained. After patient consent, tumor and normal tissues were harvested intraoperatively according to standard procedures. Routinely, the mutational status of BRAF wild-type and K, H, NRAS was monitored by Sanger sequencing. Normal, tumor, and metastatic (lymph node) tissues were collected for analysis and additionally examined by histology.

### Isolation of protein, DNA and RNA from patient tissue

Protein, DNA and RNA were isolated from the same tissue sample using AllPrep DNA/RNA/Protein Mini Kit (80,004, Qiagen) following the manufacturer’s protocol.

For Western blot analysis of proteins from patients’ tissue, samples – normal, tumor, metastasis – were lysed in 1X RIPA buffer, sonicated and centrifuged. Supernatants were mixed with Laemmli buffer, boiled and subsequently analyzed by Western blotting. Quantification was done using Image J software.

### Sequencing

RNA-seq libraries were prepared using TruSeq RNA Sample Preparation v2 kit (RS-122-2001, Illumina). Exome capture was performed using the Agilent SureSelect Human All Exome kit (50 Mb; G3370L, Agilent). RNA-seq and Exome capture libraries were sequenced on HiSeq (Illumina) to generate 2 × 150 bp and 2 × 75 bp paired-end data, respectively.

### RNA-seq data analysis

Raw sequencing reads were first trimmed to remove sequencing adapters using cutadapt (v.2.5, [6]). Next, adapter-trimmed reads were aligned to the human genome version NCBI GRCh38 [7] using STAR (v2.7.3a). Raw read counts were estimated using HTSeq (v0.11.2). Read counts were normalized using DESeq2 to get the normalized counts [8]. Fusions were identified and visualized using Arriba [9].

### cDNA isolation and PCR

cDNAs were synthesized from 500 ng of total RNA isolated from normal, tumor and metastasis samples using RevertAid First Strand cDNA synthesis kit (K1621, Thermo Fisher Scientific) and random hexamer primers. CDNAs were analyzed for the presence of fusion transcripts by PCR amplification using primers that amplify the fusion region. PCR amplification of 4 μL of cDNA (diluted 1:10 in water) was carried out using Q5 High-Fidelity DNA polymerase (New England Biolabs, M0491L), in the presence of 10 mM DNTPs, 10 μM primers in 25 μL reaction. PCR reaction was set up as described below in a 5pprox. cycler:

- Denaturation: 98°C – 30 s
- Amplification (35 cycles): 98°C – 10 s, 63/65/68°C – 30 s, 72°C – 20 s
- Extension: 72°C – 2 min

Primer sequences:

- BAIAP2L1-BRAF fwd: GTTGATAAGCACTGTGGCTT
- BAIAP2L1-BRAF rev: AGTCACAAAATGCTAAGGTG
- GAPDH fwd: CGACAGTCAGCCGCATCTT
- GAPDH rev: CCCCATGGTGTCTGAGCG
- CCDC6-RET fwd (1): CATTGTCATCTCGCCGTTC
- CCDC6-RET rev (1): GATGACATGTGGGTGGTTG
- CCDC6-RET fwd (2): CTGCAGCAAGAGAACAAGGT
- CCDC6-RET rev (2): ACCACTTTTCCAAATTCGCC
- NCOA4-RET fwd: GCTAGTTCAGCAAATATTGGG
- NCOA4-RET rev: GACGTTGAACTCTGACAGCA

PCR products were subsequently subjected to gel electrophoresis in a 1% agarose gel and visualized under UV.

For BAIAP2L1-BRAF sequencing, the 453 bp PCR product was purified using QIAquick PCR purification kit (Qiagen, 28,104) and sequenced using the following primers:

- TGTCTGGAACTCCTCAGGCTTCAC
- TGTTGATGCCATCAAAGTGCC

### Quantitative RT-PCR

cDNAs were used to quantify gene expression levels. SYBR-Green (A25780, Thermo Fisher Scientific) based qRT-PCR was performed using the StepOnePlus™ Real-Time PCR System (4376600). The expression levels were normalized to expression of GAPDH reference gene. Relative expression levels were calculated as ΔΔCt, and results presented as log2 fold change in gene expression.

Primer sequences:

- GAPDH fwd: CGACAGTCAGCCGCATCTT
- GAPDH rev: CCCCATGGTGTCTGAGCG
- BAIAP2L1-BRAF fwd: TCACCCATGATCGAGAGAAG
- BAIAP2L1-BRAF rev: TCTGTAAACAGCACAGCAC
- CCDC6-RET fwd: GCTGAAGATAGAGCTGGAGAC
- CCDC6-RET rev: ACCACTTTTCCAAATTCGCC
- NCOA4-RET fwd: TCCTTACATACCCAGCACC
- NCOA4-RET rev: GCGTTCTCTTTCAGCATCTTC
- TPO fwd: GCCTTCTTCCCCTTCATCTC
- TPO rev: GTTTCCATTATCTCTGCTGCTC
- DIO1 fwd: ACATCAGAAATCACCAGAACC
- DIO1 rev: CAGAACAGCACGAACTTCC
- IGSF1 fwd: GCTGCCACTATCTTCTCACC
- IGSF1 rev: TGTTTCTCTTTCTTCCCATTCC
- PDE5A fwd: GTTTCTTATCAGCCGCCTC
- PDE5A rev: ACACCAACAACCTCTTCCC

### Immunohistochemistry

H&E and α-thyroglobulin stainings were performed on formalin fixed paraffin embedded sections using standard laboratory procedures. Immunohistochemistry was performed on paraffin sections by using the DAKO-EnVision FLEX-kit (Dako, Glostrup, Denmark). Staining was performed on an immunostainer (Autostainer; Dako, Glostrup, Denmark) according to the manufacturer ´s instructions.

### Plasmids and constructs

pENTR221 CCDC6-RET, pENTR221 BAIAP2L1-BRAF, pENTR221 BAIAP2L1-BRAF Δ111–154, pENTR221 BAIAP2L1-BRAF kinase-dead D695A were synthesized from Thermo Fisher Scientific.

Point mutations in pENTR221 constructs were obtained by site-directed mutagenesis using Q5 High-Fidelity DNA Polymerase (Cat# MO491, New England BioLabs) and the following primers:

- CCDC6-RET kinase-dead: GTGGCCGTGATGATGCTGAA
- BAIAP2L1-BRAF DIF mutant: GAAAACACACCATGTGAATATC
- BAIAP2L1-BRAF RBD mutant: GATGATGTTAGGTCTAATCC

All pENTR221 plasmids were cloned into pDONOR221 and expression plasmids pPHAGE C-TAP (FLAG tagged), a kind gift from Prof. Dr. Christian Behrends, and pLenti-V5 DEST Vector (V5 tagged).

### Cell culture

Nthy-ori 3–1 cells (90,011,609, Sigma) were cultured in RPMI-1640 medium supplemented with 10% heat inactivated FBS at 37 °C in 5% CO_2_. HEK293T cells (a kind gift from Dr. Andreas Ernst) were cultured in DMEM supplemented with 10% heat inactivated FBS at 37 °C in 5% CO_2_. For transient transfections, cells were seeded and transfected the following day as follows: 1 μg (6-well plate) or 5 μg plasmid (100 mm dish) were mixed with 5.4 or 27 μl of 10 mM PEI (polyethylenimine, PEI 25000, Sigma-Aldrich) in 100 or 500 μl of PBS and incubated for 15 minutes at room temperature, respectively. After incubation, transfection mixture was added drop-wise onto cells. Proteins were isolated 2 days after transfection.

In order to generate Nthy-ori 3–1 cells stably expressing constructs, we first transfected HEK293T cells with pPHAGE C-TAP or pLenti-V5 DEST together with the pLenti package (HDM-VSV-G; HDM-tatlb; HDM-Hgprn2 (gag-pol); RC-CMV-Rev1b) for lentiviral particle production. After 48 h, the media containing the virus particles were sterile filtered and then added to Nthy-ori 3–1 cells in the presence of 8 μg/mL polybrene. After 48 h, cells were selected with either puromycin (2.5 μg/mL) or zeocin (100 μg/μL) and the surviving cells were expanded and maintained in puromycin or zeocin-containing media.

### Generation of 3D spheroids

Spheroids were grown in U-shaped 96-well plates (Nuncon® Thermo Fisher Scientific, #174925). Briefly, 7.500 cells were seeded in each well in RPMI supplemented with 10% FCS and in the presence of 6 μg/mL collagen. After seeding, the plate was centrifuged at 250 g for 5 minutes and the cells were incubated for 72 hours. Pictures were taken with a Leica microscope. Viability was assessed with CellTiter-Glo® 3D Cell Viability Assay (Promega, G9682) according to the manufacturer’s instructions. When needed spheroids were treated with indicated compounds for 3 more days.

### Spheroid invasion assay

4-day old spheroids were embedded in Matrigel (Corning® Matrigel® #356231). A layer of Matrigel (6 μl) was pipetted on the inner well of a μ-Slide Angiogenesis chamber (Ibidi, # 81506) and the slide was incubated at 37 °C for 5 min for polymerization. Single spheroids were placed on the center of the well and a second matrigel layer (6 μl) was added on top. Slides were incubated for 30 min at 37 °C followed by addition of 50 μl of spheroid culture medium with or without indicated compounds to each well. The invasion was monitored for 3 days before imaging.

### Imaging of spheroids

In order to determine the morphology or the invasion, spheroids were fixed with 4% paraformaldehyde (Roth, # P087.4) for 1 h at RT. Wells were washed with PBS and spheroids were stained using Hoechst (0,4 μg/ml) (Invitrogen, #H3570) and Phalloidin rhodamine (1:150) (Invitrogen, #R415) for 90 min at RT. After washing with PBS, samples were imaged using Leica TCS SP8 confocal microscope and Leica TCS SP8 DIVE multiphoton microscope in an inverted stage position with 5x and 10x objectives. Quantification was made using Imaris Software (Bitplane) by calculating the spheroid diameter on day 4 (day of matrigel embedding) and the invasion (point from the edge of day 4 spheroids to the invading branches) or regression (point from the edge of day 4 spheroids to the edge of embedded spheroids) length.

### Generation of knockdowns

For generation of TRIM25 and PKCδ knockdowns, shRNAs plasmids were purified from the MISSION® shRNA Human Library (Sigma-Aldrich) following manufacturer’s instructions. As a control the non-targeting control shRNA (shCo) MISSION® pLKO.1 puro (Cat# SHC001) was included.

For stable expression of shRNAs in Nthy-ori 3–1 cells expressing BAIAP2L1-BRAF-V5, cells were infected with lentiviral particles carrying the shRNA vectors and selected with puromycin as described in the cell culture section.

- shRNA sequences:
- shTRIM25_2:
- CCGGCCAGCTCACATCCGAACTCAACTCGAGTTGAGTTCGGATGTGAGCTGGTTTTT
- shTRIM25_3:
- CCGGGAACTGAACCACAAGCTGATACTCGAGTATCAGCTTGTGGTTCAGTTCTTTTT
- sh PKCδ_41:
- CCGGGCAAGACAACAGTGGGACCTACTCGAGTAGGTCCCACTGTTGTCTTGCTTTTT
- sh PKCδ_49:
- CCGGCAACAGCCGGGACACTATATTCTCGAGAATATAGTGTCCCGGCTGTTGTTTTTG

### Pull-down assay

Cells were seeded in 100 mm dishes and transfected with the indicated constructs. 48 h after transfection, cells were washed with ice-cold PBS and lysed with ice-cold IP buffer (10 mM HEPES pH 7.4; 150 mM NaCl, 1% Triton X-100, plus protease inhibitor cocktail Set I-Calbiochem 1:100 (Cat# 539131, Merck Millipore), 1 mM Na_3_VO_4_ and 1 mM NaF). After 30 min on ice, samples were cleared by centrifugation at 13,000 rpm for 10 min and protein concentration was measured using Pierce 660 nm Protein Assay Reagent (Cat. No. 22660, Thermo Fisher Scientific). 250 μg of protein extracts were mixed with either V5 antibody (1/50) or 20 μL of FLAG beads (ANTI-FLAG® M2 Affinity Gel, A2220-5ML, Sigma-Aldrich) and incubated for 2 hr. at 4 °C with rotating. In the case of V5 pull-down, antigen-antibody complexes were precipitated by pre-washed agarose-coupled protein A/G beads (Cat# 11134515001/11243233001, Roche) for 2 hr. at 4 °C with rotating. After incubation, beads were washed with IP buffer, mixed with Laemmli 4X buffer (0.125 M Tris-HCl, pH 6.8, 4% SDS, 10% glycerol, 10 mM DTT and bromophenol blue), boiled for 5 min at 95 °C and analyzed by Western blotting.

### In vitro kinase assay

Nthy-ori 3–1 cells were seeded in 100 mm cell culture plates and FLAG-tagged plasmids were transfected. 48 h post transfection, cells were lysed in RIPA buffer (250 mM NaCl, 50 mM Tris-HCl pH 7.5, 10% glycerol, 1% Triton X-100 with protease inhibitor cocktail) and FLAG-tagged proteins were immunoprecipitated using FLAG antibody and immobilized to agarose-coupled protein A/G beads overnight at 4 °C with rotating. Protein-bound beads were washed with the lysis buffer and used for in vitro kinase assay. The kinase assay was performed using either dephosphorylated myelin basic protein (MBP) (13–110, Merck) or inactive MEK1 K97A (M02-16H, SignalChem) as a substrate in 25 mM Tris (pH 7.5), 5 mM β-glycerolphosphate, 2 mM DTT, 0.1 mM Na_3_VO_4_, 10 mM MgCl_2_ and 5 mM ATP (Enzo, BML-EW9805–0100) in a final volume of 40 μL. The kinase assay mixture was incubated with the immunoprecipitated FLAG-tagged proteins for 30 min at 30 °C. The kinase reaction was terminated by adding 20 μL Laemmli 4X followed by boiling at 95 °C for 5 min.

### Western blot

For Western Blot analysis, cells were lysed in RIPA buffer and incubated for 30 min on ice. Samples were cleared by centrifugation at 13,000 rpm for 10 min, sonicated and protein concentration was measured. Lysates were mixed with Laemmli 4X buffer and boiled at 95 °C for 5 min. Cell lysates were loaded onto 7.5% polyacrylamide gels. The proteins were then transferred to nitrocellulose blotting membranes (10,600,001, GE Healthcare). Membranes were blocked with 3% bovine serum albumin (BSA; A7906, Sigma-Aldrich) in PBS-Tween (PBS-T) for 1 hour at room temperature and incubated with primary antibodies in 3% BSA overnight at 4 °C. Subsequently, the membranes were washed 3 times in PBS-T and incubated with horseradish peroxidase-coupled secondary antibodies for 1 h at room temperature, followed by 3 washes. The antigen–antibody complexes were detected by enhanced chemiluminescence (Immobilon Western Chemiluminescent HRP Substrate, WBKLS0500, Millipore) using Biorad ChemiDoc™ Touch Imaging System (Biorad). Quantification of Western blots was performed by using Fiji/ImageJ software (Fiji, RRID: SCR_002285).

For vemurafenib, PLX8394 and trametinib treatment, cells were serum-starved for 4 hours, treated with the indicated doses for 1 hour and harvested for Western blotting. For ponatinib treatment, cells were treated for 6 hours and harvested for Western blotting.

### NanoBiT assay

LgBit and smBit constructs were purchased from Promega. Full-length WT, G12V and S17N KRAS were cloned with *Xho* I and *Bgl* II to LgBit. BRAF WT RBD, BRAF R188L RBD and BAIAP2L1-BRAF RBD were cloned with *EcoR*I and *Bgl*II to SmBit. The constructs were transfected into HEK293T cells as following: in 12-well plates, 0,5 μg of each plasmid was transfected into cells with 0.5 mM of PEI reagent in 50 μl PBS. 2 days after transfection, cells were harvested and seeded into 96-well white plates (Greiner). After 4 hours, a Nano-Glo® Live Cell Assay (#N2012; Promega) was performed according to the manufacturer’s instructions. The luminescence was measured using a Tecan infinite reader. For PLX8394 treatment, after 4 hours incubation, cells were treated with indicated amounts of PLX8394 for 30 minutes and subsequently analyzed.

### Antibodies

In this study the following antibodies were used: Anti-thyroglobulin (M0781, Dako), anti-phospho-tyrosine (P-Tyr-1000) MultiMab™ (8954S, CST), anti-Myelin Basic Protein (MBP) (13,344, CST), anti-V5 antibody (Thermo Fisher Scientific, R960–25), anti-FLAG® M2-Peroxidase (A8592, Sigma-Aldrich), anti-phospho-RET (Y905) (3221S, CST), anti-RET (C31B4) (3223S, CST), anti-phospho-MEK1/2 (9154, CST), anti-MEK1 antibody (2352, CST), anti-phospho-p44/42 MAPK (Thr202/Tyr204) (ERK1/2) (9101 L, CST), anti-p44/42 MAPK (ERK1/2) (9102, CST), anti-actin (ab49900, abcam), anti-PKCδ (2058S, CST), anti-TRIM25 (610,570, BD Biosciences), IQGAP1 (22167–1-AP, Proteintech), anti-GAPDH (GTX627408, GeneTex).

### Soft agar colony formation assay and 3D-MTT

1.5% agarose solution was mixed with 2X growth medium (with 20% FCS, 2x inhibitor) to get a final mixture with 0.75% agarose in 1X growth medium (bottom layer). 50 μL of this bottom agar medium was added per well in a 96-well plate and incubated at room temperature for at least 10 min to solidify the agarose. The upper layer was composed of 75 μL mixture containing 25 μL 2X medium, 25 μL 1.5% agarose and 25 μL cell suspension (5000 cells). After solidification, 125 μL of 1X medium, with 2X compound if needed, was added on top. Colonies were analyzed after 5 days. In order to determine the number of colonies, cells were stained with Hoechst (0,5 mg/mL) for 30 minutes before imaging with a Leica Dmi8 Widefield (5× dry objective, Z-stack, 25 steps). The number of colonies was quantified by Fiji software using particle analyses. The number of particles from 500 μm to infinity (500 μm–∞) was quantified. 3D-MTT assay was performed using Cell Proliferation Kit I (11,465,007,001, Roche). Briefly, 25 μl of the MTT solution was added into each well and incubated for 3 h at 37 °C. Afterwards, the medium was removed, and 175 μl of solubilization buffer was added. The plate was incubated at 70 °C for ~ 1 h until the agar melted. The content of the wells was gently mixed and transferred into a new 96-well plate, the absorbance of the solubilized MTT was measured at 570 nm with a Tecan infinite reader. For each condition, the average of three replicates was calculated. Then, the percentage of viable cells was determined as: Percentage of viable cells = 100*(OD of experiment- OD of the background)/(OD of control-OD of the background).

### Heteromerization studies

Cells were seeded in 6-well plates and transfected with BAIAP2L1-BRAF-FLAG-expressing plasmid. Forty-eight after transfection, cells were treated either with DMSO (A3672.0250, Applichem), dithio-bis-maleimidoethane (DTME) alone (0.2 mM, 1 h; 22,335, Thermo Fisher Scientific) or DTME followed by Dithiothreitol (DTT) (100 mM, 15 min). After thorough washes with PBS, cells were lysed in sample buffer (cells treated with only DTME were lysed in non-DTT-containing sample buffer) and subjected to immunoblot analysis.

### Filter-aided sample preparation (FASP)

For mass spectrometric analysis, samples were processed using a modified filter-aided sample preparation (FASP) protocol. In brief, samples dissolved in a urea-based buffer (7 M urea, 2 M thiourea, 5 mM dithiothreitol (DTT), 2% (w/v) CHAPS) were lysed by sonication at 4 °C for 15 min using a Bioruptor (Diagenode, Liège, Belgium). The protein concentration was determined using the Pierce 660 nm protein assay (Thermo Fisher Scientific) according to the manufacturer’s protocol. Proteins (corresponding to 20 μg) were transferred onto spin filter columns (Nanosep centrifugal devices with Omega membrane, 30 kDa MWCO; Pall, Port Washington, NY). Afterwards, detergents were removed washing the samples (membrane) three times with a buffer containing 8 M urea. After reduction and alkylation by DTT and iodoacetamide (IAA), excess IAA was quenched with DTT and the membrane washed three times with 50 mM NH_4_HCO_3_. Afterwards, proteins were digested overnight at 37 °C with trypsin (Trypsin Gold, Promega, Madison, WI) using an enzyme-to-protein ratio of 1:50 (w/w). After digestion, peptides were recovered by centrifugation and two additional washes with 50 mM NH_4_HCO_3_. Combined flow-throughs were acidified with trifluoroacetic acid (TFA) to a final concentration of 1% (v/v) TFA and lyophilized. Purified peptides were reconstituted in 0.1% (v/v) formic acid (FA) for LC-MS analysis.

### Liquid chromatography-mass spectrometry (LC-MS)

Tryptic peptides were analyzed by LC-MS on a Synapt G2-S HDMS mass spectrometer (Waters Corporation) coupled to a nanoAcquity UPLC system (Waters Corporation). Water containing 0.1% (v/v) FA, 3% (v/v) dimethyl sulfoxide (DMSO) served as mobile phase A and acetonitrile (ACN) containing 0.1% FA (v/v), 3% (v/v) DMSO as mobile phase B (PMID: 23975139). Peptides (corresponding to 200 ng) were loaded in direct injection mode onto an HSS-T3 C18 1.8 μm, 75 μm × 250 mm reverse-phase column (Waters Corporation). Separation of peptides was conducted at a flow rate of 300 nL/min running a gradient from 5 to 40% (v/v) mobile phase B over 90 min. Afterwards, the LC column was washed with 90% mobile phase B and re-equilibrated to initial conditions resulting in a total analysis time of 120 min. The column was heated to 55 °C. Eluting peptides were analyzed in positive mode ESI-MS by ion-mobility separation (IMS) enhanced data-independent acquisition (DIA) UDMS^E^ mode. Acquired MS data were post-acquisition lock mass corrected using [Glu1]-Fibrinopeptide B, which was sampled every 30 s into the mass spectrometer via the reference sprayer of the NanoLockSpray source at a concentration of 250 fmol/μL.

LC-MS DIA raw data were processed and searched with ProteinLynx Global SERVER (PLGS) (version 3.02 build 5, Waters Corporation) against a custom compiled database containing UniProtKB/SwissProt entries of the human reference proteomes (entries: 20,365) as well as common contaminants. Following search criteria were applied: (i) Trypsin as digestion enzyme allowing up to two missed cleavages, (ii) carbamidomethyl cysteine was defined as fixed and (iii) methionine oxidation as variable modification. The false discovery rate (FDR) for peptide and protein identification was assessed searching a reversed database and set to a 1% threshold for database search in PLGS. Label-free quantification analysis was performed using ISOQuant. For each protein, absolute in-sample amounts were estimated using TOP3 quantification.

The mass spectrometry proteomics data have been deposited to the ProteomeXchange Consortium (http://proteomecentral.proteomexchange.org) via the jPOST partner repository with the dataset identifiers PXD034229 (ProteomeXchange) and JPST001610 (jPOST).

### Integration of RNA-seq and proteomics data

Differential expression analysis was performed using DeSeq2 (v1.6) to identify 1214 significantly deregulated genes (adjusted *p*-value < 0.05 and log2 fold change > or < 0). This list of genes was queried against the 1127 deregulated proteins from the proteomics data to identify 20 commonly significantly deregulated genes in the 4 fusion-carrying patients using the intersect function in the R (v3.3.2) package dplyr (v1.0.2). Volcano plots of genes/proteins commonly deregulated or of genes/proteins only deregulated in either RNA-seq or proteomics data, respectively, were plotted using the R package ggplot2 package (v3.3.3).

### Statistical analysis

Data were statistically analyzed by using GraphPad Prism software. Tests employed are mentioned in the figure legends.

## Results

**Fig. 1:**
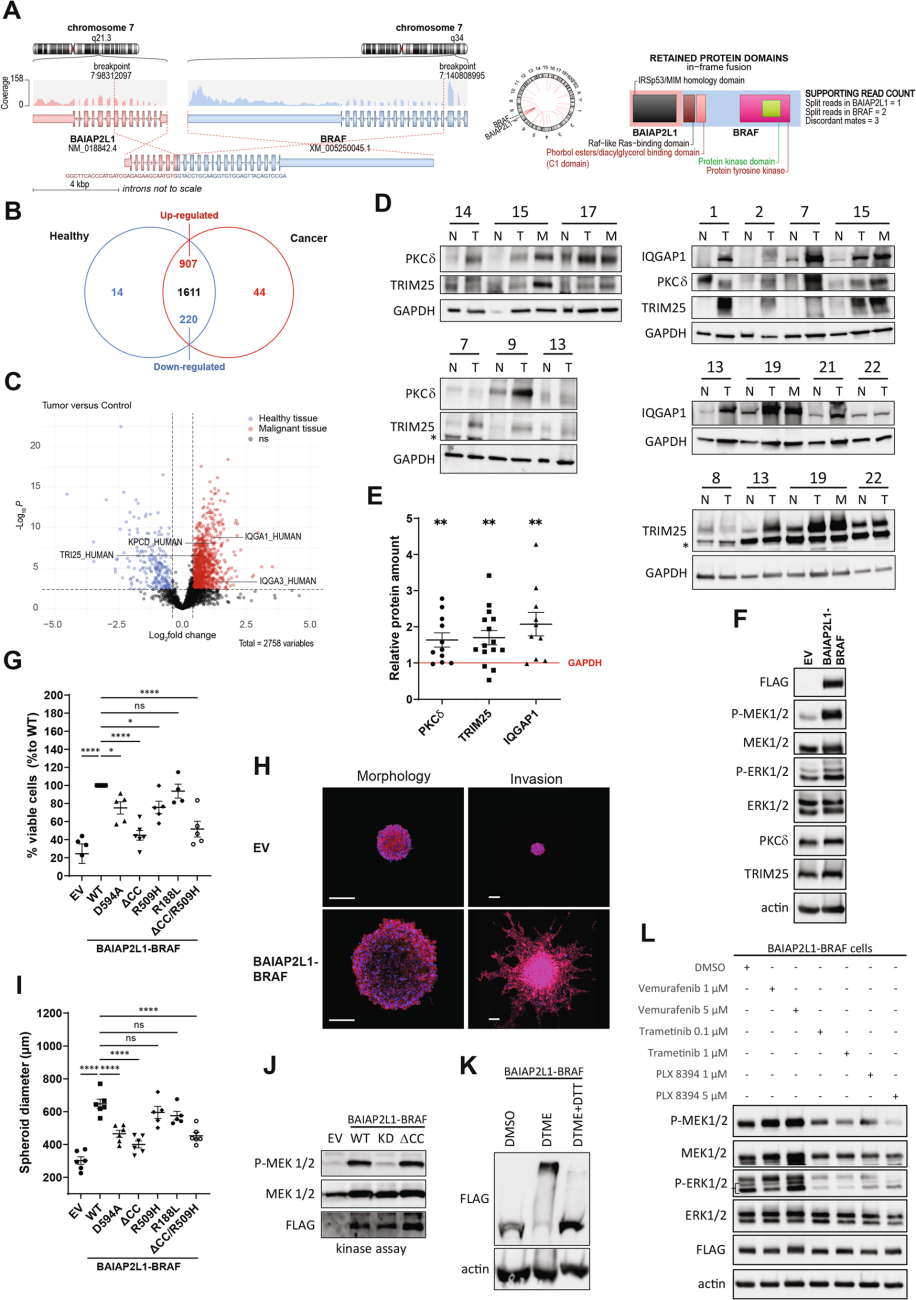
A new BRAF fusion BAIAP2L1-BRAF was detected in a PTC patient. **A** New BAIAP2L1-BRAF fusion revealed in patient 17 by RNAseq analysis. **B** Venn diagram and **C.** Volcano plot representing the differential expression of proteins detected in the mass spectrometric data from normal tissues compared to the corresponding tumor lesions of 4 patients (#14, #15, #17, #21) (Benjamini-Hochberg corrected t-test, *p* > 0.01, log2(fold change) > 0.4, detected in at least 9 LC-MS runs). **D** Western blot detection of TRIM25, PKCδ and IQGAP1 in normal and tumor/metastasis tissues of several patients of an additional cohort. **E** Relative quantification of PKCδ *n* = 11), TRIM25 (*n* = 15) and IQGAP1 (*n* = 10) protein levels obtained from Western Blot analysis of various patients’ protein extracts (Tumor and Metastasis vs Normal) (error bars = SEM, paired t test, two-tailed, *P* value: ** < 0.01). GAPDH was used for normalization. **F** MAPK pathway activation detected by Western blotting of proteins extracted from Nthy-ori 3–1 cells transiently transfected with EV and BAIAP2L1-BRAF WT. **G** 3D MTT of Nthy cells stably expressing EV, BAIAP2L1-BRAF-FLAG WT or different BAIAP2L1-BRAF mutants after colony formation assay (error bars = SEM, *n* = 5, *n* = 4 for R188L, Dunnett’s multiple comparisons test, *P* value: * < 0.033, **** < 0.0001, ns-not significant). **H** Fluorescence microscopy images of Hoechst (blue)/phalloidin (red) staining of spheroids from cells stably expressing EV and BAIAP2L1-BRAF-FLAG WT 3 days after seeding (left) or embedded in Matrigel for 72 h (right). Scale bar: 200 μm. **I** Same as in G, but quantification of the spheroid diameters. Spheroids of Nthy cells stably transfected with EV, BAIAP2L1-BRAF-FLAG WT or BAIAP2L1-BRAF-FLAG mutants were analyzed (error bars = SEM, *n* = 6, Dunnett’s multiple comparisons test, *P* value: **** < 0.0001, ns-not significant). **J** Kinase activity assay of Nthy-ori 3–1 cells transiently transfected with EV, BAIAP2L1-BRAF WT, kinase-dead (KD) or coiled-coil domain deleted fusion (ΔCC) expressing plasmids. **K** Western blot detection of a crosslinking and release experiment using DTME and DTME+DTT, respectively. Nthy-ori 3–1 cells were transiently transfected with BAIAP2L1-BRAF-FLAG WT plasmid and treated for 1 hour with DTME or DMSO and for 15 minutes with DTT. **L** MAPK pathway detected by Western blotting of proteins extracted from Nthy-ori 3–1 cells of cells stably expressing BAIAP2L1-BRAF-FLAG WT, treated for 1 hour with DMSO, vemurafenib, trametinib or PLX-8394 with indicated doses. Representative Western blots are shown

In our effort to identify molecular changes that drive tumorigenesis in PTCs, we collected materials of a cohort of 22 consenting PTC patients following our cancer center’s standard operating procedures (SOPs) and initially focused on 4 patients, including a minor (#14, #15, #17, #21), for further detailed analysis based on their clinical parameters (Suppl. Fig. 1A, B). Further, all the 4 patients were devoid of RAS and BRAF mutations in their tumour samples during the initial analysis. All patients underwent total thyroidectomy and two patients are pre-operatively diagnosed with thyroiditis. Patient #14 is diagnosed with mild chronic Thyroiditis and patient #17 is diagnosed with Hashimoto Thyroiditis and elevated thyroglobulin antibodies. The samples were quality controlled by qualified pathologists and authenticated by the expression of validated biomarkers including α-thyroglobulin as exemplified for the 4 patients shown in Suppl. Fig. 1A. Protein, DNA, and RNA were isolated as described in the methods section and subjected to comprehensive omics analysis including RNA-seq, quantitative proteomics as well as an integrative analysis of RNA-seq and proteomics. By RNA-seq analysis, we identified 3 different gene fusions in the 4 patients of the cohort including two known RET fusions: CCDC6-RET in patient 14 as well as in patient 15, and NCOA4-RET in patient 21 (Suppl. Fig. 1C-E). In a female patient (#17) with Hashimoto’s thyroiditis we found a fusion that had not been described before. It was an intrachromosomal fusion (chromosome 7) where the 5′ region of the BAIAP2L1 was fused to the BRAF kinase leading to a BIAP2L1-BRAF fusion with an intact kinase domain and most of the RAS-binding domain (RBD) (Fig. 1A). Using RT-PCR primers specific for the fusion product, we unexpectedly detected it in both tumor and adjacent normal tissue, even though to a lesser extent, suggesting that it was probably already present in the inflamed tissue (Suppl. Fig. 1F). To rule out the possibility that the fusion was a germline event, we examined the PBMCs from the patient’s blood. In these samples we could not detect the fusion product (Suppl. Fig. 1F).

In order to reveal factors that are deregulated in the tumor tissue compared to the matching normal tissue, we first performed RNA sequencing analysis of the 4 patients mentioned above (Suppl. Fig. 2A). In total we identified the corresponding protein-coding RNAs of 18,378 targets, of which 646 were significantly upregulated (adjusted *p*-value < 0.05 and log2 fold change > 0) and 568 significantly downregulated (adjusted *p*-value < 0.05 and log2 fold change < 0) in the tumor tissue (supplementary Table 1). Initially, we selected a panel of thyroid-related factors based on the existing literature (please see supplementary Table 2 for the list with the references), and screened our RNAseq data for these factors. Consistent with the published observations, many of these factors were significantly downregulated and only a few were upregulated in the patients’ tumor tissues (Suppl. Fig. 2B). Because the normal tissue of patient 17 corresponds more to an inflamed tissue due to the diagnosed Hashimoto’s thyroiditis, we analyzed the expression differences detected in this patient independently of the other three patients. However, with a few exceptions, the selected thyroid-related factors were comparably regulated between the three patients and patient 17 (Suppl. Fig. 2B). Most of these factors were undetectable in the proteomic analysis (see next paragraph), so no conclusions can be drawn about regulatory mechanisms at the protein level. We additionally confirmed the differentially expression of TPO and DIO1 seen in the RNAseq (Suppl. Fig. 2C) by qPCR (Suppl. Fig. 2D). Of note, compared to the other 3 patients, patient 17 exhibited significantly reduced RNA levels of TPO and DIO1 already in the normal tissue (Suppl. Fig. 2E) confirming noteworthy differences between the normal tissues of the selected patients.

To identify factors that were dysregulated at the protein level in tumors compared with matched normal tissue, we then performed quantitative label-free proteomics on the same patient materials (Fig. 1B, C and Suppl. Fig. 3A). We detected 2817 proteins in total. Of these, 14 proteins occurred exclusively in the healthy samples and 44 occurred exclusively in tumor tissue (supplementary Table 3). One thousand six hundred eleven of them showed no changes in their expression level, 907 were upregulated and 220 were downregulated in the tumor. With a main focus on proteins involved in the cell signaling machinery, we identified, among the upregulated candidates, kinases (e.g., PKCδ), components of the ubiquitination machinery (e.g., TRIM25), and adaptor proteins (e.g., IQGAP1) (Fig. 1C, Suppl. Fig. 3B, C). Western blot analysis of additional patients from the cohort confirmed the upregulation of PKCδ, TRIM25, and IQGAP1 on the protein level in the tumor and metastasis samples of several patients in comparison to the matching normal tissue (Fig. 1D, E). Since BRAF V600E mutation was detected in patient 7 and patient 13, our data suggest that deregulation of these factors is not unique to fusion-bearing PTCs. Unlike PKCδ and TRIM25, which showed no regulation at the RNA level, IQGAP1 RNA was upregulated in three out of four patients (Suppl. Fig. 3D).

Finally, we performed integrative analysis of RNA-seq and proteomics data using the list of differentially expressed genes and their corresponding proteins, respectively, from the 4 fusion-carrying patients to identify factors significantly deregulated at both the mRNA and protein levels. This analysis yielded 20 identified factors (Suppl. Figs. 4 and 5), including phosphodiesterase 5 A (PDE5A) and immunoglobulin superfamily member 1 (IGSF1, also called p120), a factor known to be associated with hypothyroidism, which are upregulated in tumor and/or metastatic tissue in comparison to the matching normal tissue (Suppl. Fig. 4B). We further verified the observed regulation of PDE5A and IGSF1 by RT-PCR (Suppl. Fig. 4C). Of note, the tumor sample from patient 17 showed the upregulation of PDE5A and IGSF1 rather on the protein, but not on the RNA level, as opposed to other patients.

We next characterized the previously unknown BRAF fusion product identified in patient 17, by overexpressing the BAIAP2L1-BRAF protein in human immortalized thyroid cells. As expected, expression of the wild-type fusion led to the activation of the MEK1/2-ERK1/2 cascade (Fig. 1F). In the proteomic analysis, we observed only a marginal increase in the levels of TRIM25 and PKCδ for patient 17. The reason could be that the “normal” tissue corresponded to inflamed tissue due to Hashimoto’s thyroiditis in this patient. Consequently, we were interested to see how the expression of BAIAP2L1-BRAF affected TRIM25 and PKCδ levels compared with control cells, and under these conditions we indeed verified a slight increase in their protein levels (Fig. 1F).

To evaluate the oncogenicity of the fusion, we performed 3D MTT assays and soft agar colony formation assays. Indeed, expression of BAIAP2L1-BRAF significantly increased cell proliferation (3D MTT assay, Fig. 1G) and colony formation (soft agar assay, Suppl. Fig. 6B) of the cells. We then attempted to recapitulate the in vivo scenario using 3D spheroids stably expressing BAIAP2L1-BRAF to subsequently investigate their invasion ability (Fig. 1H, I). Compared to the empty vector control, not only the size of the spheroids, but also the invasive phenotype was greatly increased when BAIAP2L1-BRAF was expressed (Fig. 1H, I), thus underlining the transforming ability of the BRAF fusion. We additionally generated BAIAP2L1-BRAF mutants potentially interfering with the function of the fusion and tested for their ability to induce colony formation or invasion of the cells. Therefore, either the BRAF kinase domain (corresponding to BRAF_D594A; “kinase dead”), the RAF dimerization interphase (corresponding to BRAF_R509H) or the RAS binding domain (corresponding to BRAF_R188L) was mutated, the coiled-coil (CC) domain of BAIAP2L1 (ΔCC) was deleted or the R509H and ΔCC mutations were combined (Suppl. Fig. 6B). As shown in Fig. 1G and Suppl. Fig. 6A, all mutations except of the R188L mutation had a significant impact on the ability of Nthy cells to form colonies compared to Nthy cells transfected with the original fusion product. However, the expression of the ΔCC mutation reduced the ability to form colonies the most. Regarding spheroid size and invasion, transfection of the kinase dead or ΔCC mutant had the greatest impact (Fig. 1H, I and Suppl. Fig. 6C-E). Interestingly, expression of BAIAP2L1-BRAF-R509H or R188L mutants in Nthy cells did not significantly affect spheroid size (Fig. 1I) and even resulted in a mild invasive phenotype (Suppl. Fig. 6C-E). However, invasion was still greatly reduced compared with the invasion induced by the wild type BAIAP2L1-BRAF fusion (Suppl. Fig. 6C-E). We then analyzed the ability of the various BAIAP2L1-BRAF mutants to activate the MEK1/2-ERK1/2 pathway and observed that the ability to activate MEK1/2 was impaired after overexpressing the fusion protein carrying the D594A (kinase dead, KD), R509H or ΔCC mutation (Suppl. Fig. 7A). In contrast, mutation of the RAS-binding domain (R188L) did not impair the ability of the fusion protein to activate MEK1/2 (Suppl. Fig. 7A). Interestingly, although the ΔCC mutant strongly inhibited MEK1/2 phosphorylation in stably transfected Nthy cells (Suppl. Fig. 7A), it didn’t affect the kinase activity of the BAIAP2L1-BRAF fusion in transient transfected cells (Fig. 1J). Next, we performed cross-linking experiments that showed that BAIAP2L1-BRAF was multimerized in cells (Fig. 1K), for which both the BRAF kinase domain, known to dimerize to be active, and the coiled-coil domain (CC) of BAIAP2L1 may be responsible. In dimerization experiments, we examined the interaction between V5-tagged BAIAP2L1-BRAF WT and different FLAG-tagged BAIAP2L1-BRAF variants. While the R509H mutation had a significant impact, deletion of the coiled-coil domain did not interfere with the dimerization of the proteins (Suppl. Fig. 7B). In addition, we could not detect consistent differences in the interaction of the BAIAP2L1-BRAF mutants with endogenous RAFs (Suppl. Fig. 7C). We further tested whether the novel fusion protein, in which the RBD domain was not completely preserved, bound the RAS protein. By pulldown assays using the lysates of Nthy-ori 3–1 cells transiently overexpressing V5-tagged BAIAP2L1-BRAF WT and FLAG-tagged KRAS G12V, we demonstrated that KRAS G12V in general interacted with the WT fusion protein (Suppl. Fig. 7D). The interaction was significantly impaired by the R188L mutation within the Ras binding domain of BAIAP2L1-BRAF WT, but not by the R509H mutation (Suppl. Fig. 7D). We next determined the interaction between the RBD domain of BRAF WT, the RAS-binding deficient RBD domain (R188L) of BRAF or the RBD domain of BAIAP2L1-BRAF with KRAS by a NanoBiT assay (Suppl. Fig. 7E). We detected a strong interaction of the BRAF WT RBD domain with KRAS WT and KRAS G12V as expected, but not with KRAS S17N, proving the functionality of the performed NanoBit assay. In line with this, mutation of the BRAF RBD (R188L) resulted in a remarkably reduced interaction with KRAS. Interestingly, the NanoBit assay further revealed that the interaction between the RBD domain of BAIAP2L1-BRAF with KRAS is strongly impaired compared to the interaction seen with BRAF WT (Suppl. Fig. 7E). Together with the phenotypical characterization of BAIAP2L1-BRAF-transformed Nthy cells, our data suggest that the coiled-coil domain as well as kinase activity are critical for BAIAP2L1-BRAF fusion function, whereas RAF dimerization and RAS binding may contribute but play a minor role in the oncogenic process.

**Fig. 2:**
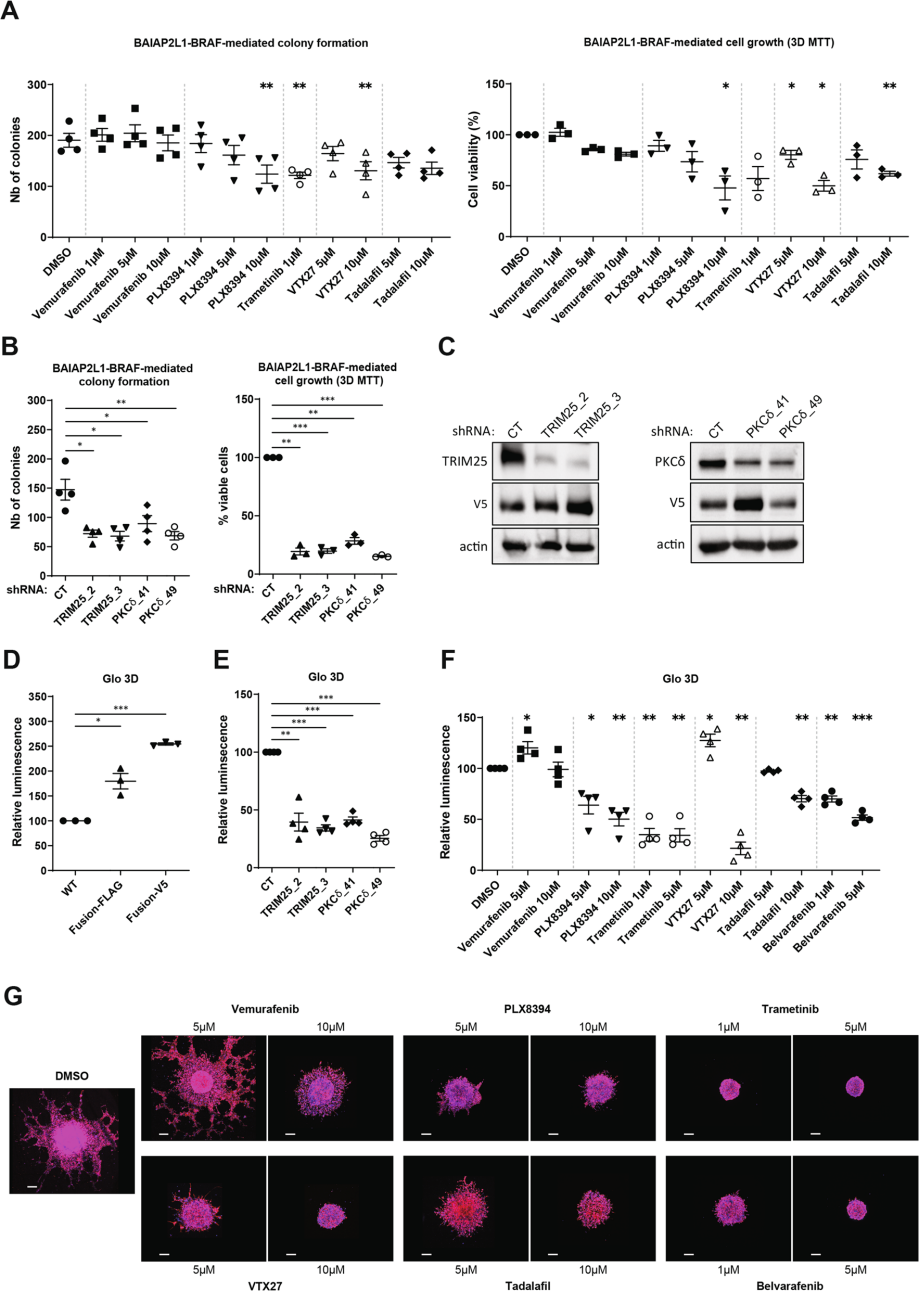
Inhibition of BRAF or factors deregulated in tumors blocks BAIAP2L1-BRAF-mediated cell transformation. **A** Colonies counts (left, *n* = 4) and 3D MTT (right, *n* = 3) of cells stably expressing BAIAP2L1-BRAF-FLAG WT and analysed 5 days after indicated treatments (error bars = SEM, paired t test, two-tailed, *P* value: * < 0.05, ** < 0.01). **B** Colonies counts (left, *n* = 4) and 3D MTT (right, *n* = 3) of cells stably expressing BAIAP2L1-BRAF-V5 WT and the indicated shRNAs, analysed 5 days after seeding (error bars = SEM, paired t test, two-tailed, *P* value: * < 0.05, ** < 0.01, *** < 0.0001). **C** Western blot detection of extracts from Nthy-ori 3–1 cells stably expressing BAIAP2L1-BRAF-V5 WT and the indicated shRNAs. **D** Nthy-ori 3–1 cells stably expressing EV, BAIAP2L1-BRAF-FLAG or BAIAP2L1-BRAF-V5 and **E** Nthy-ori 3–1 cells stably expressing BAIAP2L1-BRAF-V5 along with indicated shRNAs were cultured as 3D spheroids for 3 days in the presence of 6 μg/mL collagen. Presence of metabolically active cells was measured with 3D viability Glo Assay (error bars = SEM, *n* = 3 or 4, paired t test, two-tailed, *P* value: * < 0.05, ** < 0.01, *** < 0.0001). **F** Nthy-ori 3–1 cells stably expressing BAIAP2L1-BRAF-FLAG were cultured as 3D spheroids for 3 days, treated with indicated compounds and incubated for 3 more days. Cell viability was assessed with 3D viability Glo Assay (error bars = SEM, *n* = 4, paired t test, two-tailed, *P* value: * < 0.05, ** < 0.01, ns-not significant). **G** Representative fluorescence microscopy images of Hoechst (blue)/phalloidin (red) staining of spheroids from cells stably expressing BAIAP2L1-BRAF-FLAG WT embedded in Matrigel for 3 days and treated with indicated compounds. Scale bar: 200 μm

We then tested the ability of various inhibitors to reduce MAPK signaling in fusion-expressing cells (Fig. 1L). Treatment with the BRAF inhibitor vemurafenib at lower concentrations led to the well-documented “paradoxical” activation of the MAPK pathway [10]. However, the BRAF dimer-breaker PLX8394 inhibited MAPK signaling with comparable efficiency to the MEK1/2 inhibitor trametinib. We confirmed by NanoBit assays that PLX8394 inhibits the dimerization of BAIAP2L1-BRAF protein in a concentration-dependent manner (Suppl. Fig. 7F). Thus, our data suggest that RAF dimer-breakers may serve an effective treatment strategy for tumor patients with this BRAF fusion. We then tested whether BAIAP2L1-BRAF-mediated transformation could be prevented with inhibitors targeting the factors we have identified by our multiomic approach. Indeed, treatment with inhibitors of PDE5A (tadalafil) and PKCδ (VTX27) reduced both colony formation and growth of the cells to a similar extent as inhibitors of BRAF (PLX8934) and of MEK (trametinib) (Fig. 2A). We confirmed these observations for PKCδ by knockdown studies with shRNAs (Fig. 2B). Additionally, we induced shRNA-mediated knockdown of TRIM25 in BAIAP2L1-BRAF-transformed cells and demonstrated that this reduced colony formation of fusion-transformed thyroid cells as well (Fig. 2B). Efficient knockdown of PKCδ and TRIM25 was confirmed by Western Blotting (Fig. 2C). Next, we employed our 3D spheroid model with Nthy cells stably expressing BAIAP2L1-BRAF and determined the viability and growth of spheroid cultures by a 3D Glo assay. Indeed, we confirmed our initial observation from Fig. 1H, I and demonstrate that the size of spheroids formed by BAIAP2L1-BRAF transformed Nthy cells was enhanced compared to the Nthy control cells (Fig. 2D, Suppl. Fig. 8A). Consistent with our previous findings, depletion of TRIM25 or PKCδ in BAIAP2L1-BRAF transformed Nthy cells reduced the viability and growth of 3D spheroid cultures (Fig. 2E, Suppl. Fig. 8B). Similar results were obtained when spheroids were treated with inhibitors of BRAF (PLX8934 and belvarafenib), PKCδ (VTX27), or PDE5A (tadalafil) (Fig. 2F, Suppl. Fig. 8C). Next, we performed spheroid invasion assays and screened the aforementioned inhibitors for their potential to inhibit invasion of BAIAP2L1-BRAF-transformed Nthy cells. As illustrated in Fig. 2G and Suppl. Fig. 9A, B, all inhibitors indeed had a significant effect on it. These data suggest that these factors could potentially be targeted to treat patients who carry the BAIAP2L1-BRAF gene fusion.

Since we found those factors deregulated in other patients as well we also analyzed the effect of their inhibition on cells expressing the most common RET fusion CCDC6-RET which was here identified in patient 14 and 15. First, we confirmed that expression of the CCDC6-RET fusion in Nthy-ori 3–1 cells enhanced their colony formation in soft agar which could be reduced by mutation of the RET kinase domain (CCDC6-RET KD) (Suppl. Fig. 9C, D). Analysis of extracts of Nthy-ori 3–1 cells transiently expressing the WT fusion protein showed that MEK1/2 was activated and the levels of TRIM25 and PKCδ were elevated (Suppl. Fig. 9E). The enhanced TRIM25 and PKCδ are in line with our proteome data demonstrating a deregulation of these factors in patient samples (Fig. 1B, C; Suppl. Fig. 3B, C). We then compared the effect of inhibiting PKCδ (VTX27), PDE5A (tadalafil), and CCDC6-RET (ponatinib) on colony formation and viability of fusion-expressing Nthy-ori 3–1 cells and found that all three inhibitors significantly reduced both features (Suppl. Fig. 9F). Ponatinib, approved by the FDA for patients with chronic myeloid leukemia and Philadelphia chromosome-positive acute lymphoblastic leukemia, is known for its multi-targeted characteristics and inhibits not only the tyrosine kinase BCR-Abl, but also other tyrosine kinases such as FGFR, PDGFR, SRC, RET, KIT, and FLT1. We additionally verified the efficiency of ponatinib against the CCDC6-RET fusion by Western blot analysis (Suppl. Fig. 9G).

## Discussion

The shift towards a more personalized therapeutic approach involving the use of molecular characterization began only about two decades ago. The obvious advantage of precision medicine is that therapeutic targets can be used for treatment that depend on the characteristics of the specific tumor, rather than simply using nonspecific cytotoxic drugs. In addition, precision medicine can help match the aggressiveness of treatment to the risk of the disease. This is beneficial, among others, in tumor diseases such as PTC, where overall survival is generally good, but aggressive forms still occur, posing a major therapeutic challenge. For instance, the identification of RET fusion proteins has contributed to the use of selective RET inhibitors for therapy, which have been shown to be effective while having lower systematic toxicity than nonspecific tyrosine kinase inhibitors. There is a clear need to continue and expand tumor profiling to identify additional therapeutic targets useful for patient-tailored therapies.

In our study, in which we performed multiomics analysis using patient materials from a cohort of patients, we identified an atypical BRAF fusion carrying a partial RBD in addition to already known RET fusions. A BAIAP2L1-BRAF fusion has been described in 2016 in spitzoid melanoma [11], however, both breakpoints were found to be different than in the investigated PTC patient: 1) BAIAP2L1 breakage resulted in a truncated protein after exon 8 and 12 (conserving SH3 domain) in patient #17 and melanoma, respectively and 2) BRAF breakage resulted in a truncated protein starting at exon 4 (with a partial RBD) and 9 (lacking completely RBD) in patient #17 and melanoma, respectively. Thus, the BAIAP2L1-BRAF fusion found in spitzoid melanoma is a more “classical” BRAF fusion. Indeed, the great majority of oncogenic BRAF breakpoints result in a protein starting between exons 9 and 11, thus lacking RBD domain. However, we verified the transforming ability of this new BRAF fusion in immortalized human thyroid cells.

The tumor sample with the BRAF fusion came from a patient suffering from Hashimoto‘s thyroiditis. Although we detected the BRAF fusion also in the matching normal tissue we excluded a germline event by which the fusion could have arisen. Consequently, we hypothesize that the occurrence of the BRAF fusion in inflamed tissue may have been one of the initial events leading to tumorigenesis, or at least that the BRAF fusion contributed to the development of PTC. Although it is already known that Hashimoto’s thyroiditis is linked to a higher risk of cancer, the potential drivers or biomarkers that promote or occur during tumor development from inflamed tissue have not been well described. However, some correlations between Hashimoto’s thyroiditis and thyroid cancer have been already reported. For instance, concurrent Hashimoto’s thyroiditis was associated with the BRAF V600E mutation [12]. Interestingly, a study determining BRAF expression levels in a cohort of patients without BRAF V600E mutations revealed an enhanced BRAF expression in benign specimens with Hashimoto’s thyroiditis compared to samples without thyroiditis [13]. Thus, it would be clearly warranted to screen more patients with Hashimoto’s thyroiditis in order to identify tumor drivers that occur in the inflamed as well as in the tumor tissue.

We additionally characterized the fusion in more detail and evaluated how the functional domains of the fused proteins contribute to the overall function of the fusion product. BAIAP2L1 possesses a coiled-coil (CC) domain, which is in general important for protein interactions. Deletion of the coiled-coil domain in the identified BAIAP2L1-BRAF fusion strongly affected its ability to induce oncogenic transformation in Nthy cells, although the kinase activity of BRAF and the ability to dimerize was not affected. Thus, how BAIAP2L1 contributes to the overall function of the fusion remains to be elucidated. In contrast to the ΔCC mutant, our study revealed that mutating the RAF dimerization interphase (R509H) or the RAS binding domain (R188L) did impair the oncogenic transformation of Nthy cells, but to a much lesser extent. In addition, RAS-induced activation apparently plays only a minor in the activation of the BRAF fusion. Intriguingly, analyzing the downstream signaling events revealed that the stable expression of the ΔCC mutant, but not the R188L mutant, inhibited MEK1/2 activation and one can speculate that oligomerization mediated by the CC domain might have a clear functional implication here. Thus, whether the fusion partner BAIAP2L1 is required to induce BRAF activation deserves more investigation. For instance, BAIAP2L1 might induce a more stable RAF dimerization interphase, as it has been reported for some RAF inhibitors [14], promoting the active state of the BRAF kinase. Alternatively, feedback loops or crosstalk with other signaling pathways might play a role, which might also explain why the stable expression of the ΔCC mutant did not activate MEK1/2, although its kinase activity was not impaired. It is indeed interesting to functionally characterize the interactome of the fusion protein.

Finally, our study has uncovered not only the novel BAIAP2L1-BRAF fusion but also other druggable candidates such as TRIM25, PKCδ, and PDE5A, which appear to be more common targets as they are deregulated independently of the discovered fusion. TRIM25, an E3 ligase enzyme, has been already proposed as a therapeutic target for other cancer types such as breast cancer [15] and hepatocellular carcinoma [16]. In our previous publication, we also described that several members of the ubiquitin signaling machinery were deregulated in PTC samples [17], which again fits with the emerging development of drugs targeting the ubiquitin signaling machinery. The serine/threonine kinase PKCδ has been described to be implicated in several cellular processes such as cell proliferation, cell death and survival [18]. In fact, depending on the apoptotic stimulus, it has been reported that PKCδ functions either as a pro- or antiapoptotic factor [18]. The same is true in the context of tumorigenesis, with studies reporting both tumor-suppressive and tumor-promoting functions [18]. Interestingly, in case of PDE5A, researchers are interested in expanding the use of the inhibitors, which have been approved for erectile dysfunction, to other indications such as heart disease, diabetes, and cancer.

Consistent with the demonstrated deregulation of PKCδ and PDE5A in tumor samples, our study showed that PKC or PDE5A inhibitors inhibited colony formation and spheroid growth of Nthy-ori cells transformed with either BAIAP2L1-BRAF or CCDC6-RET fusion. In case of the BAIAP2L1-BRAF fusion RAF dimer breakers have been proven to be effective as well, again highlighting the functional importance of the dimerization of the fusion. Moreover, RAF dimer breakers, as well as PKCδ or PDE5A inhibitors, inhibited the invasion of BAIAP2L1-BRAF transformed Nthy-ori cells, which is an important feature of tumor growth and thus an important clinical link. Our study demonstrates once again the potential of multiomic analysis to discover novel targets and how the integrative analysis leads not only to the identification of patient-specific targets, but also targets that are present in a group of patients. This, in turn, is very useful to potentially draw on a larger repertoire of already approved inhibitors, which can then be tailored to the patient.

## Conclusions

Multiomic analyses are important to identify and characterize targets, which in turn can advance precision medicine and personalized therapeutics. It is important to describe not only new targets but also established ‘druggable’ targets in order to have access to a broad range of treatment strategies. Thus, the treatment regimes could be improved by employing already approved inhibitors in a way that targets the molecular profile of the patients tumor at various levels.

## Supplementary Information

**Additional file 1: Suppl. Fig. 1.** RET fusions were identified in 3 patients. A. H&E and α-thyroglobulin staining of indicated patients’ tumor tissues. B. Summary of patients’ features. C. Circos plots showing RET fusions detected in patients 14, 15 and 21. D./E. RET fusions detection by qPCR (E) and RT-PCR (F) in indicated samples. F. BAIAP2L1-BRAF detection by qPCR (upper panel) and RT-PCR (lower two panels) in the indicated patients’ samples. The upper graph shows three technical replicates. **Suppl. Fig.** **2****.** Numerous thyroid-related genes are dysregulated in tumor tissue compared to normal tissue. A. Heat map of RNA-seq analysis of normal vs tumor tissue of the 4 patients carrying the fusion proteins (adjusted *p*-value < 0.05, log2 fold change ≠ 0). B. Comparison of RNA-seq data of 19 thyroid-related genes. Patient 17 is here compared to “all patients” representing the three pateints 14, 15 and 21. C. Individual normalized read counts of 2 thyroid-related genes, TPO and DIO1. D. Determination of the relative expression of TPO and DIO1 in tumor and metastasis vs normal samples of all four patients by qPCR. Three technical replicates of each patient are shown (error bars = SEM). E. Determination of the relative expression of TPO and DIO1 in normal patient 17 vs other patients’ normal samples by qPCR (error bars = SEM, three technical replicates). **Suppl. Fig. 3.** TRIM25 and PKCδ are not upregulated at the mRNA level. A. Heat map of mass spectrometry analysis of normal vs tumor vs metastasis. B. Relative protein intensities from mass spectrometric data of PKCδ, TRIM25 and IQGAP1 from the individual patients. C. Differential expression (log2(fold change)) of PKCδ, TRIM25 and IQGAP1 from mass spectrometry data in patients #14, #15, #17 and #21. Each dot represents the log2(fold change) of one patient. D. RNA-seq data showing PKCδ, TRIM25, and IQGAP1 expression in indicated samples. **Suppl. Fig. 4.** Combination of mass spectrometric vs RNA-seq data allowed identification of additional targetable factors. A. Table summarizing factors that are up- or downregulated on both the RNA and protein levels. B. Relative protein intensities from mass spectrometric data (left) or normalized read counts from RNA-seq data (right) of IGSF1 and PDE5A in the indicated samples. C. qPCR analysis of PDE5A and IGSF1 expression in indicated samples (error bars = SEM, three technical replicates shown). **Suppl. Fig. 5.** Combination of mass spectrometric vs RNA-seq data allowed identification of additional targetable factors. Scatter plots showing significantly differentially expressed RNA (blue), protein (green) and both (red) in patients 14, 15, 17 and 21 (healthy vs tumor). **Suppl. Fig. 6.** 3D growth and invasion of Nthy cells stable transfected with BAIAP2L1-BRAF mutants. A. Representative images of the colony formation assay that has been quantified in Fig. 1F. B. Schematic illustration of WT BAIAP2L1-BRAF fusion (WT aminoacids highlighted in green) and mutated BAIAP2L1-BRAF fusions (modified residues highlighted in red) used in this study. C. Same as in Fig. 1G, but representative images of BAIAP2L1-BRAF wildtype and mutants. Scale bar: 200 μm. D. Quantification of matrix invasion of spheroids (shown in C) from cells stably expressing BAIAP2L1-BRAF-FLAG WT after treatment with indicated compounds. E. Same as in D, but the mean invasion of four independent experiments is plotted relative to the WT control (error bars = SEM, Dunnett’s multiple comparisons test, *P* value: ** < 0.002, **** < 0.0001). **Suppl. Fig. 7.** MAPK activation depends on BAIAP2L1-BRAF DIF but not on CC domain or RAS activation status. A. MEK1/2 activation detected by Western blotting of proteins extracted from Nthy-ori 3–1 cells stable transfected with the indicated plasmids (B-BRAF = BAIAP2L1-BRAF). B. Western blot detection of a V5 pull-down experiment done with extracts from Nthy-ori 3–1 cells transiently transfected with the indicated constructs (B-BRAF = BAIAP2L1-BRAF). C. Nthy cells were transiently transfected with the indicated FLAG-tagged BAIAP2L1-BRAF constructs. The empty vector served as a control. A FLAG pull down was performed and the extracts were analzyed for the presence of endogenous RAF proteins by Western blot analysis (B-BRAF = BAIAP2L1-BRAF). D. Western blot detection of a FLAG pull-down experiment done with extracts from Nthy-ori 3–1 cells transiently transfected with the indicated constructs (B-BRAF = BAIAP2L1-BRAF). E. Nanobit experiment of HEK cells transiently transfected with LgBiT-N KRAS constructs and SmBiT-C RBD constructs (error bars = SEM, *n* = 3, paired t test, two-tailed, *P* value: ** < 0.01, *** < 0.0001, ns-not significant). F. NanoBit experiment of HEK cells transiently transfected with SmBiT-N BAIAP2L1-BRAF + LgBiT-C BAIAP2L1-BRAF constructs and treated for 1 hour with increasing doses of PLX839 (error bars = SEM, *n* = 3). **Suppl. Fig. 8.** Targeting PKCδ, PDE5A, or TRIM25 sensitizes 3D spheroids stably expressing BAIAP2L1-BRAF fusion. A. Nthy-ori 3–1 cells stably expressing EV, BAIAP2L1-BRAF-FLAG or BAIAP2L1-BRAF-V5 and B. Nthy-ori 3–1 cells stably expressing BAIAP2L1-BRAF-V5 along with indicated shRNAs were cultured as 3D spheroids for 3 days in the presence of 6 μg/mL collagen. **C.** Nthy-ori 3–1 cells stably expressing BAIAP2L1-BRAF-FLAG were cultured as 3D spheroids for 3 days, treated with indicated compounds and incubated for 3 more days. **Suppl. Fig. 9.** CCDC6-RET expressing cells are sensitive to PKCδ and PDE5A inhibitors. A. Quantification of matrix invasion of spheroids (shown in Fig. 2G) from cells stably expressing BAIAP2L1-BRAF-FLAG WT after treatment with indicated compounds. B. Same as in A, but the mean invasion of four independent experiments is plotted relative to the DMSO control (error bars = SEM, paired t test, two-tailed, *P* value: * < 0.05, ** < 0.01, ns-not significant). C. Colony counts of Nthy-ori 3–1 cells stably expressing the indicated constructs (error bars = SEM, *n* = 3, paired t test, two-tailed, *P* value: * < 0.05). D. Kinase assay performed with extracts from Nthy-ori 3–1 cells transiently transfected with indicated constructs. Representative Western blot is shown. E. Representative Western blot analysis of protein extracts from Nthy ori cells transiently transfected with EV or CCDC6-RET-FLAG plasmids. F. Colonies counts (left) and 3D MTT (right) of cells stably expressing CCDC6-RET-FLAG WT analysed 5 days after indicated treatments (error bars = SEM, *n* = 3, paired t test, two-tailed, *P* value: * < 0.05, ** < 0.01). G. Western blot analysis of protein extracts from Nthy-ori 3–1 cells stably expressing CCDC6-RET-FLAG and treated with either DMSO or ponatinib for 6 hours. Representative Western blot is shown.

**Additional file 2: Suppl. Table 1.** List of differentially expressed RNAs identified in the RNAseq analysis of patients #14, #15, #17 and #21. Differentially expression of protein-coding RNAs are highlighted in the second sheet.

**Additional file 3: Suppl. Table 2.** List of thyroid-related factors. Based on the literature, a panel of thyroid-related factors were selected.

**Additional file 4: Suppl. Table 3.** Quantitative mass spectrometry analysis of normal, tumor and metastasis tissue of patients #14, #15, #17 and #21. Proteins exclusively detected in healthy tissue or malignant tissue are highlights in the second excel sheet.

## Data Availability

The data are available from authors upon request. The mass spectrometry proteomics data have been deposited to the ProteomeXchange Consortium (http://proteomecentral.proteomexchange.org) via the jPOST partner repository (doi: 10.1093/nar/gkw1080) with the dataset identifiers PXD034229 (ProteomeXchange) and JPST001610 (jPOST). Sequencing data were submitted to the EBI ENA database under accession id XX (Pending) and are available in the supplement.

## Abbreviations

ALK: Anaplastic Lymphoma Kinase
AGK: Acylglycerol Kinase
BCR-ABL: Breakpoint Cluster Region-Abelson murine leukemia viral oncogene homolog 1
BRAF: v-Raf murine sarcoma viral oncogene homolog B1
CCDC6-RET: coiled-coil domain-containing protein 6-Rearranged during Transfection
CC: Coiled-coil
DNA: Deoxyribonucleic acid
DIO1: Deiodinase 1
ERK1/2: Extracellular signal-regulated kinase 1/2
FGFR: Fibroblast growth factor receptor
FLT1: Fms-like tyrosine kinase-1
IQGAP1: IQ motif-containing GTPase activating protein 1
IGSF1: immunoglobulin superfamily member 1
LCK: Lymphocyte-specific kinase
LN: lymph node
MAPK: Mitogen-Activated Protein Kinase
MEK: Mitogen-activated protein kinase kinase
MTT assay: 3-(4,5-dimethylthiazol-2-yl)-2,5-diphenyl-2H-tetrazolium bromide assay
NCOA4-RET: Nuclear Receptor Co-Activator 4-Rearranged during Transfection
NTRK: Neurotrophic tyrosine receptor kinase
PBMC: Peripheral blood mononuclear cells
PDE5A: phosphodiesterase 5 A
PDGFR: Platelet-derived growth factor receptor
PKCδ: Protein kinase C delta isoform
PTC: Papillary thyroid carcinoma
RAS: Rat Sarcoma virus
RBD: RAS-binding domain
RET: Rearranged during Transfection
RNA: ribonucleic acid
SOP: standard operating procedures
TRIM25: tripartite motif containing 25
TPO: Thyroid peroxidase
shRNA: short hairpin RNA
SRC: Sarcoma
WT: wild-type
