# Supplementary material for: Multiomic analysis of papillary thyroid cancers identifies BAIAP2L1-BRAF fusion and requirement of TRIM25, PDE5A and PKCδ for tumorigenesis
Source: Mol Cancer. 2022 Oct 10;21:195. doi: 10.1186/s12943-022-01665-y (PMC9549631; doi:10.1186/s12943-022-01665-y)

Suppl. Figure 1

A

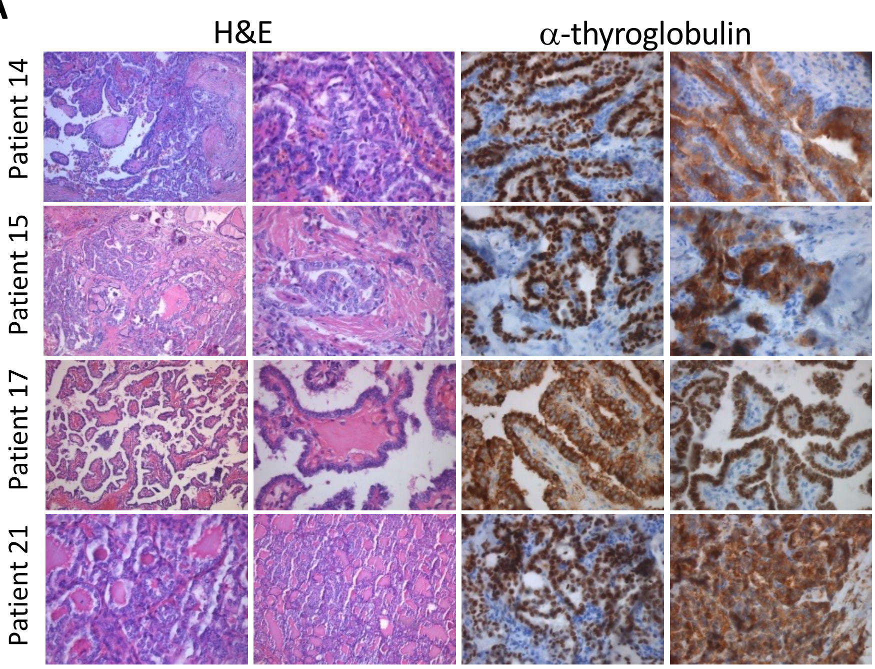

F

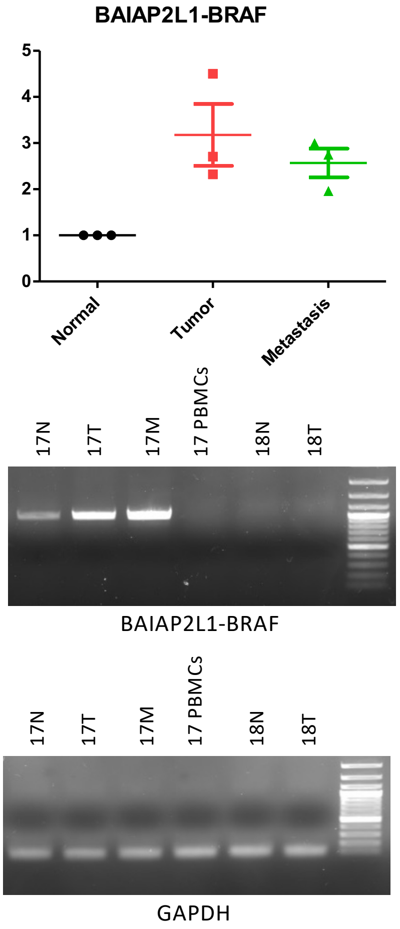

B

Pateint #	F/M	age	available samples	BRAF V600E	RAS	treatment	TNM classification
Patient 14	M	30	normal / tumor	No	No	total thyroidectomy and central lymph node dissection	pT1b, pN1b (4/17)
Patient 15	F	22	normal / tumor / metastasis	No	No	total thyroidectomy and central lymph node dissection	pT1b, pN1b (4/17)
Patient 17	F	23	normal / tumor / metastasis	No	No	total thyroidectomy and central lymph node dissection	pT1b, pN1b (6/9)
Patient 21	M	7	normal / tumor	No	No	total thyroidectomy and central and left lateral lymph node dissection (metastases in both compartments)	pT2, pN1b (7/47)

C

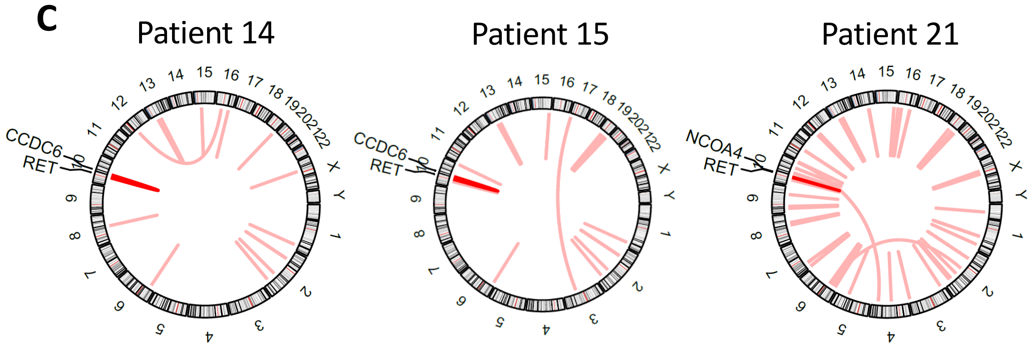

E

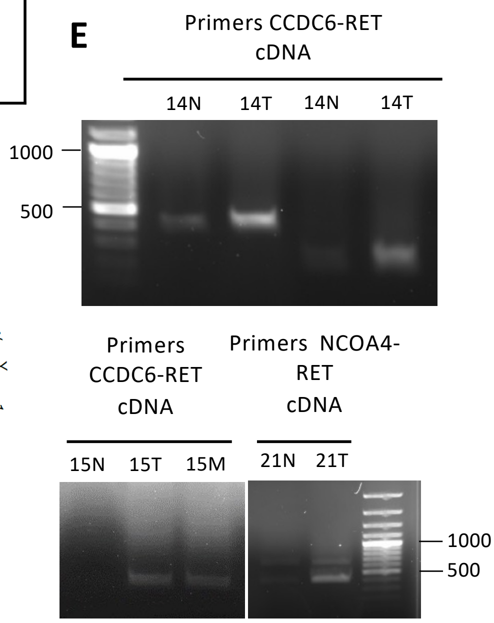

D

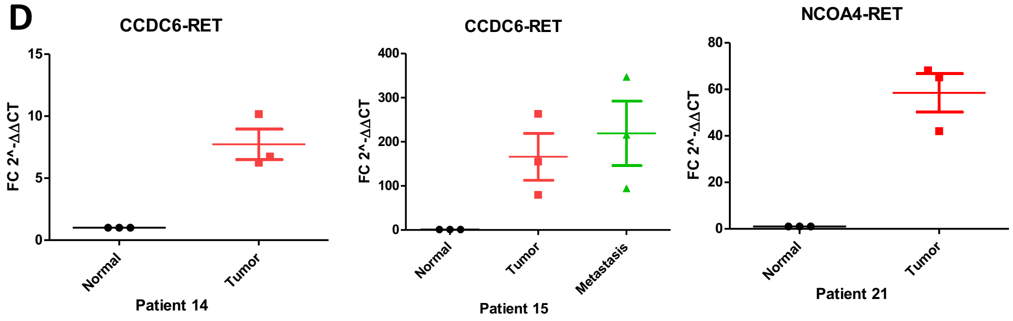

Suppl. Figure 2

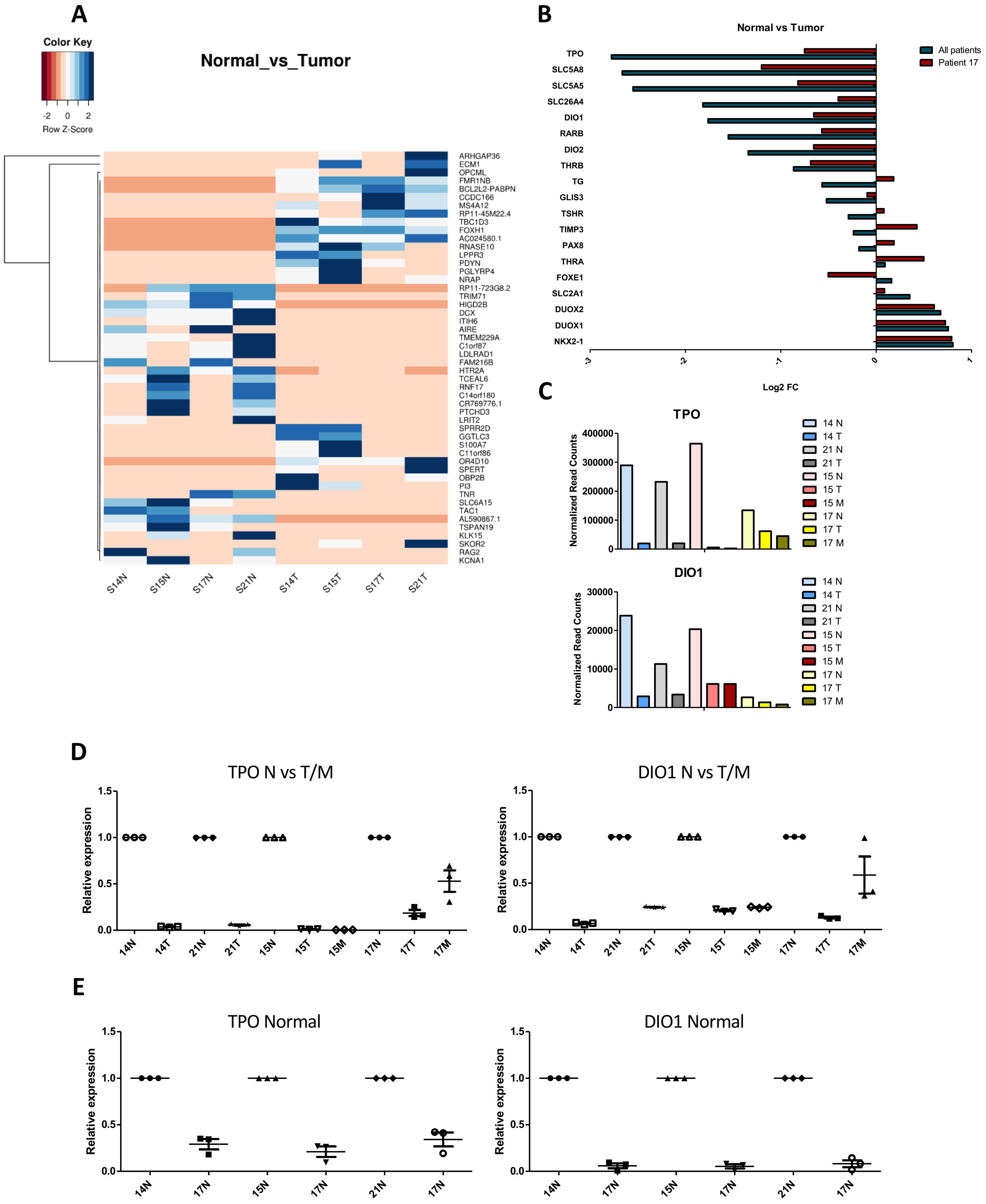

Suppl. Figure 3

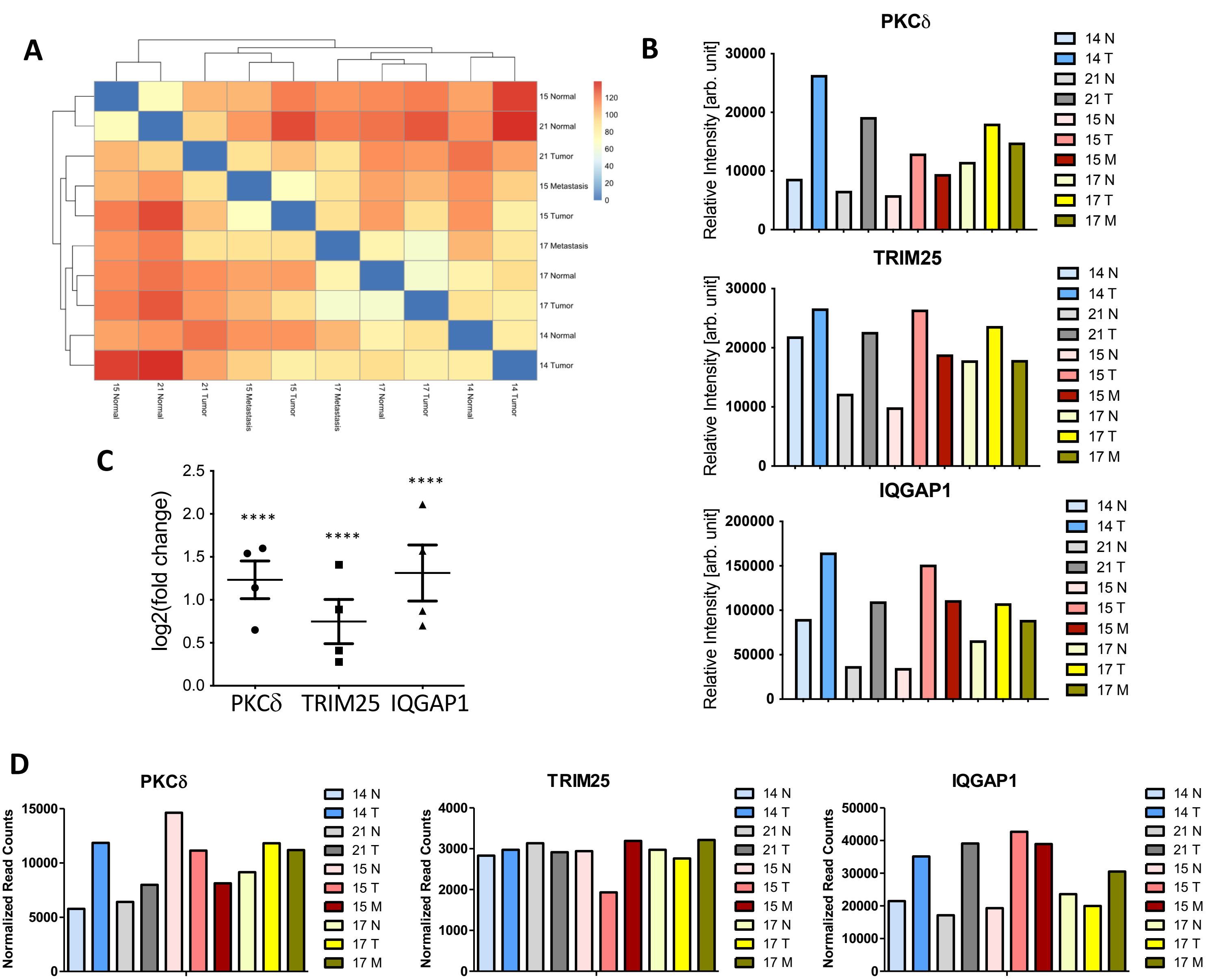

Suppl. Figure 4

A

Up-regulated		Down-regulated	
**ARG2**	14-15-21	**ELMO1**	15-21
**BID**	15-21	**FABP4**	15-21
**CBLN1**	14-21	**MFAP4**	14-21
**DPP4**	14-15-21	**TFF3**	15-21
**ECM1**	15-21		
**HMGA2**	14-15-21		
**ICAM1**	15-21		
**ISGF1**	14-15-17-21		
**MRC2**	15-21		
**MYL4**	15-17		
**MYO1G**	15-21		
**NASPA**	14-15		
**PDE5A**	14--15-21		
**PRR15**	15-21		
**RXRG**	14-21		
**TIMP1**	15-17-21		

B

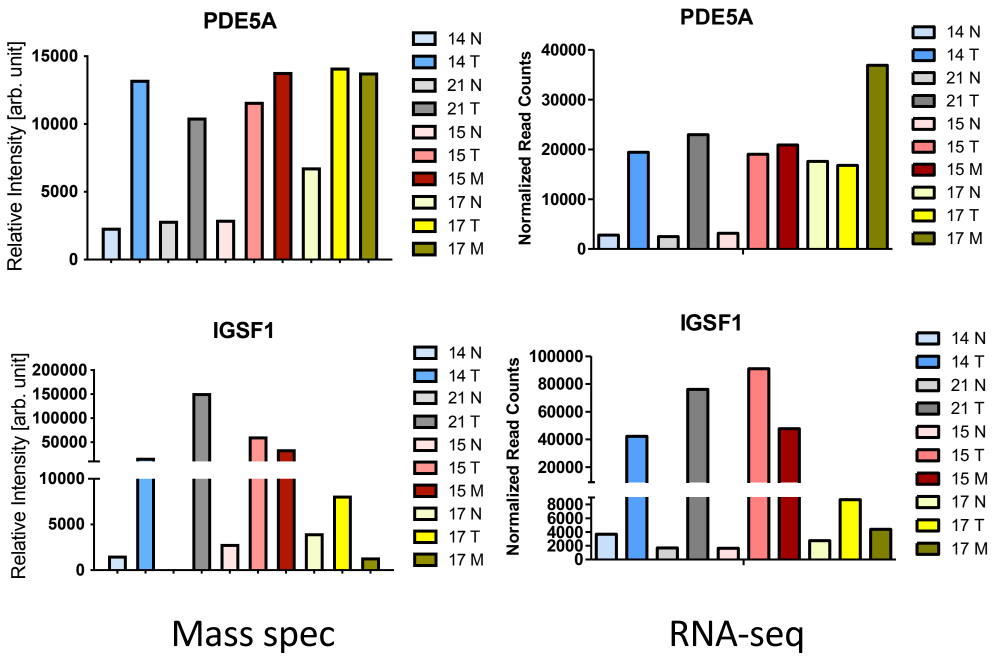

C

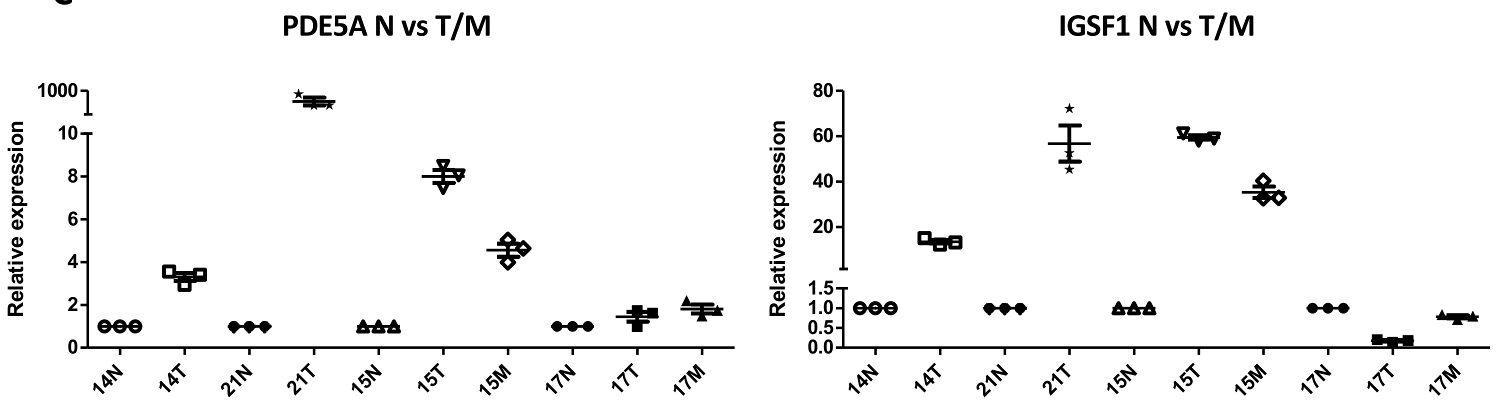

Suppl. Figure 5

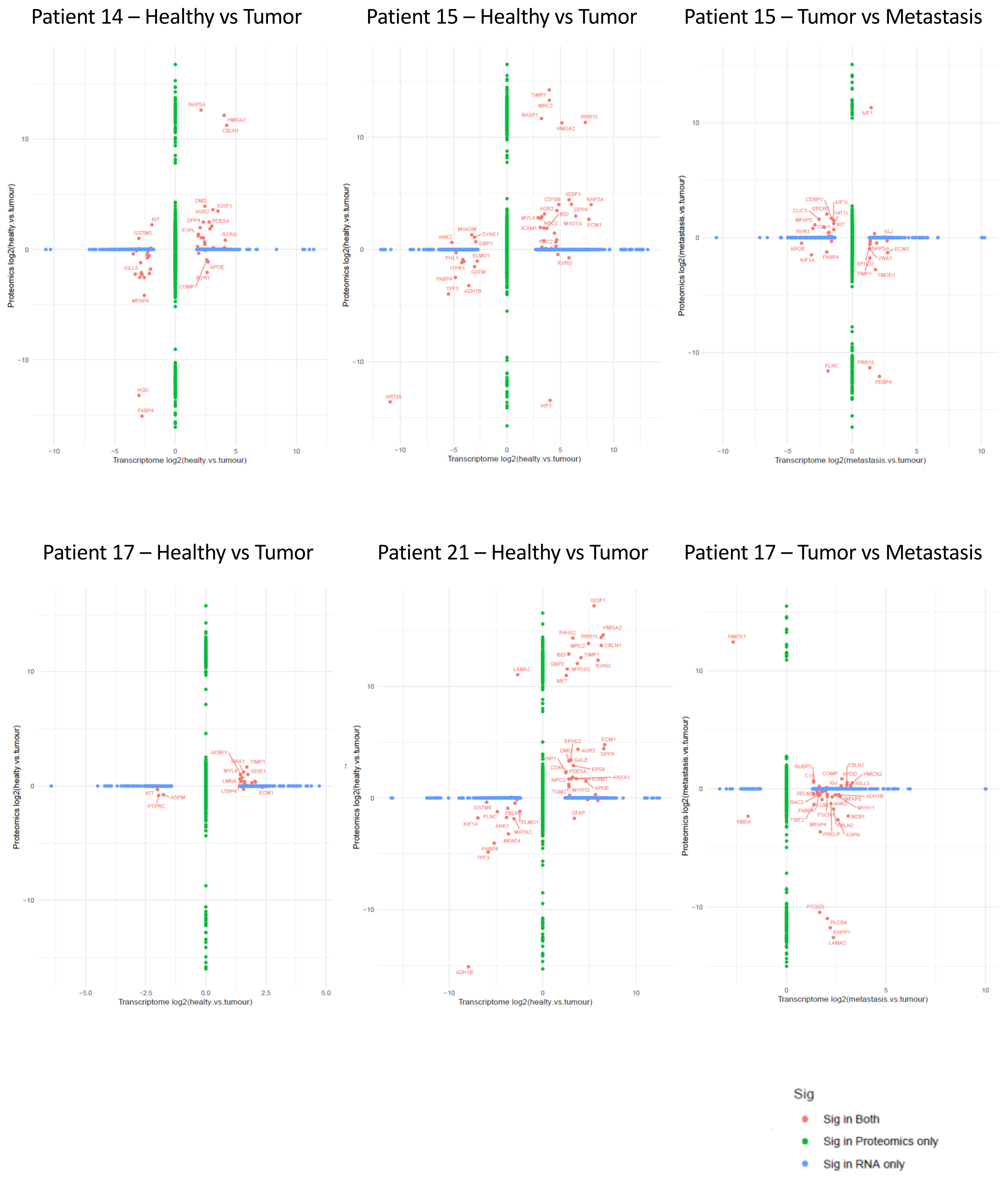

Suppl. Figure 6

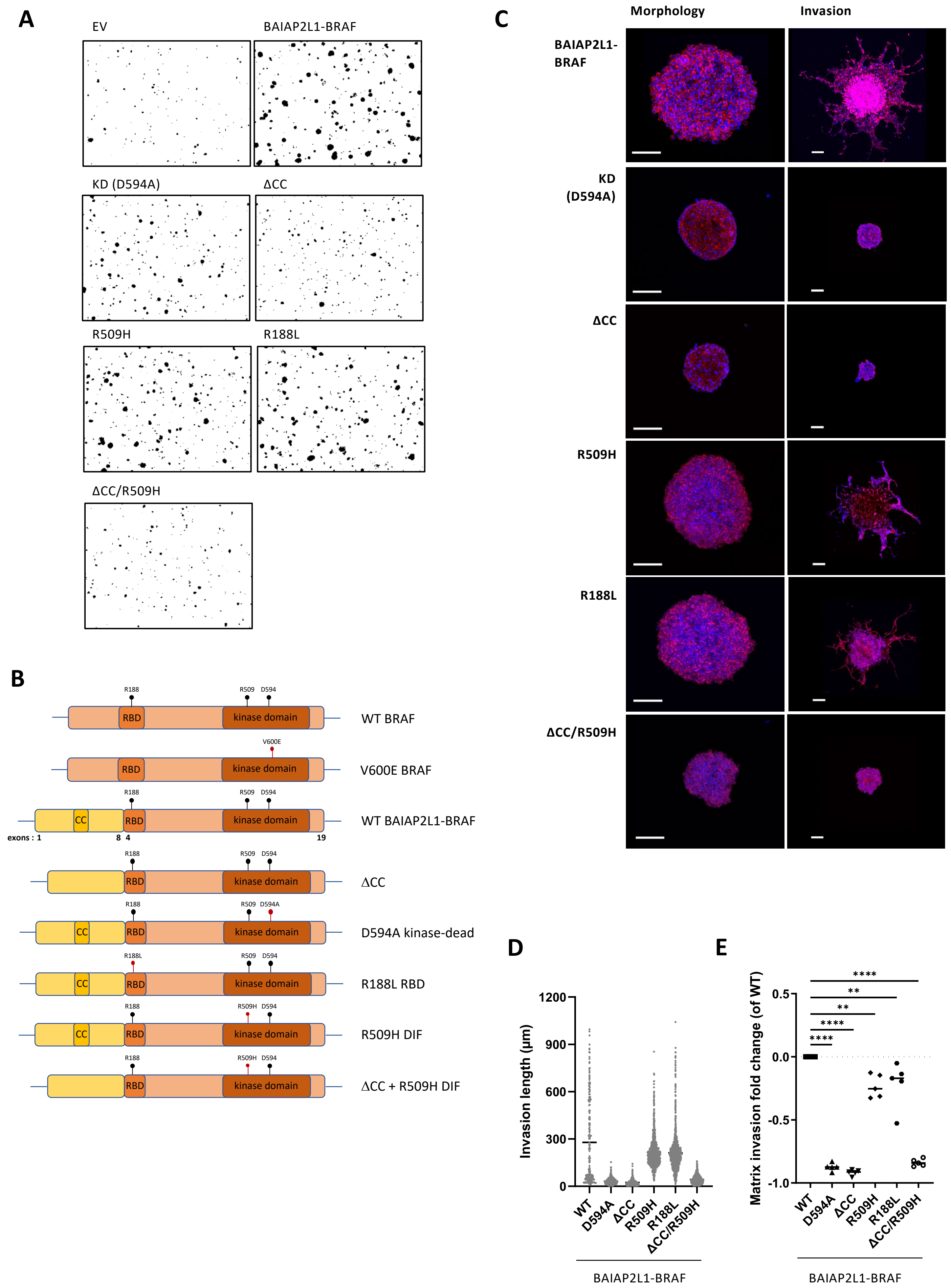

Suppl. Figure 7

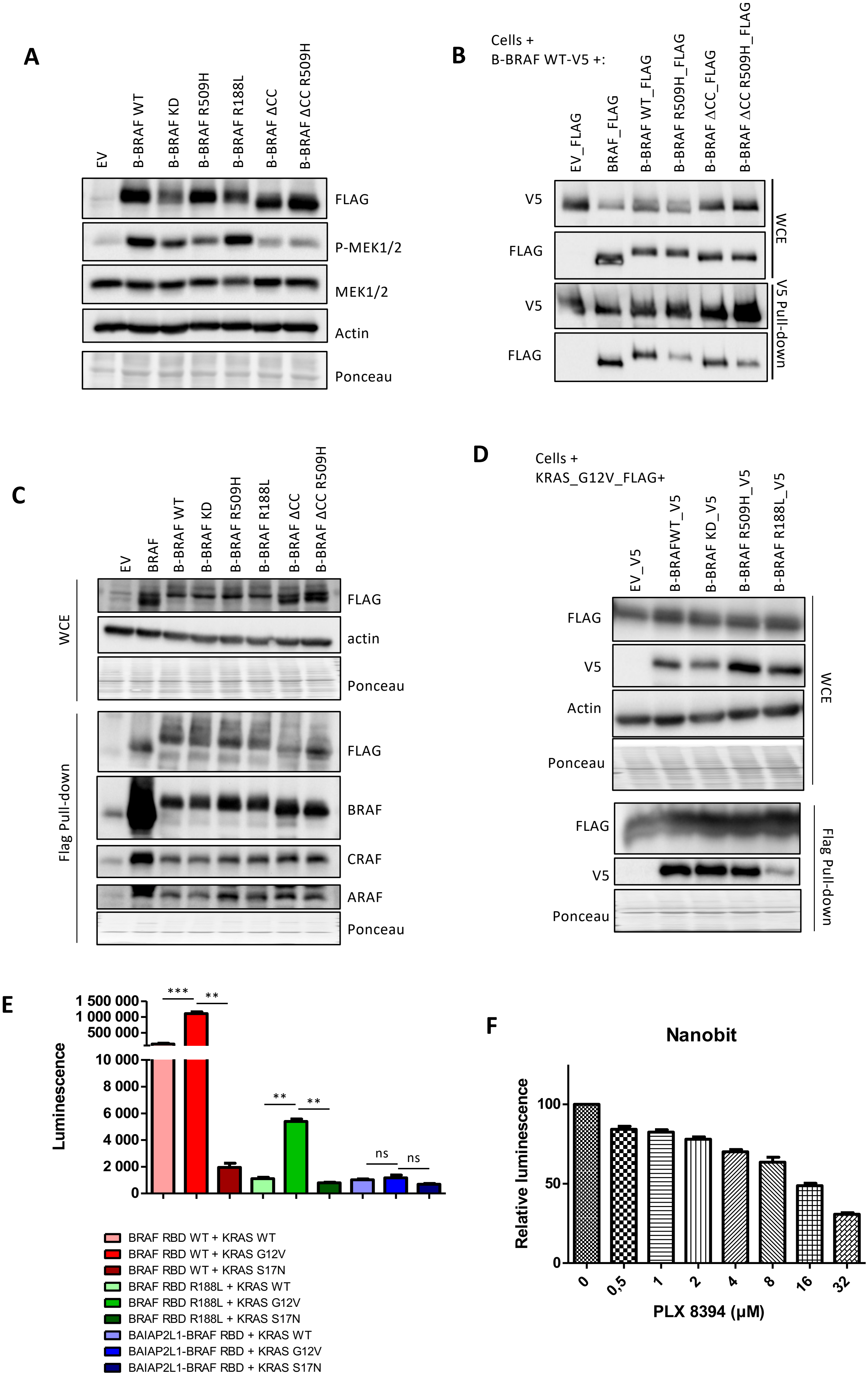

Suppl. Figure 8

A

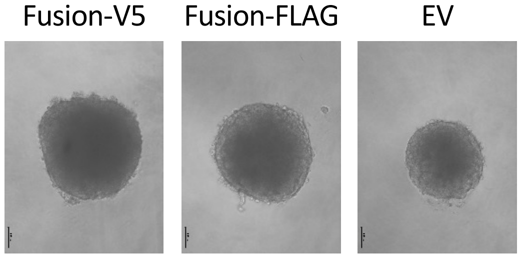

B

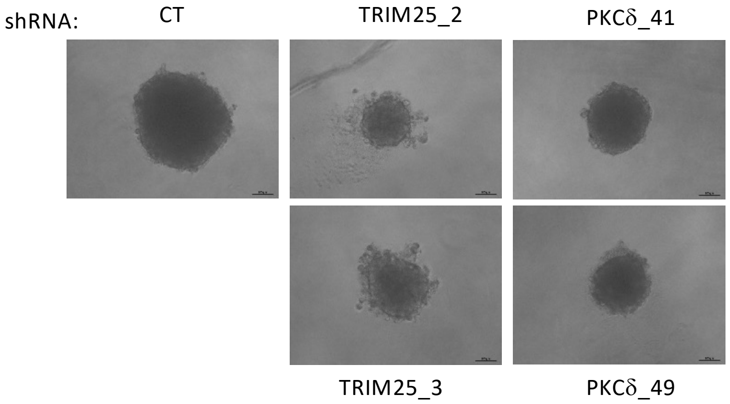

C

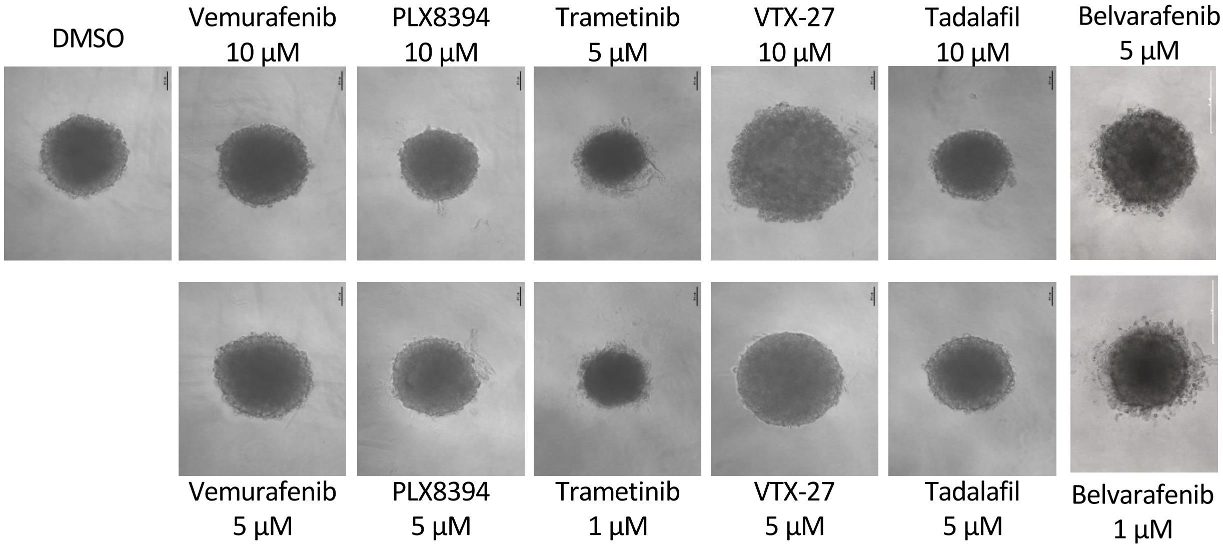

Suppl. Figure 9

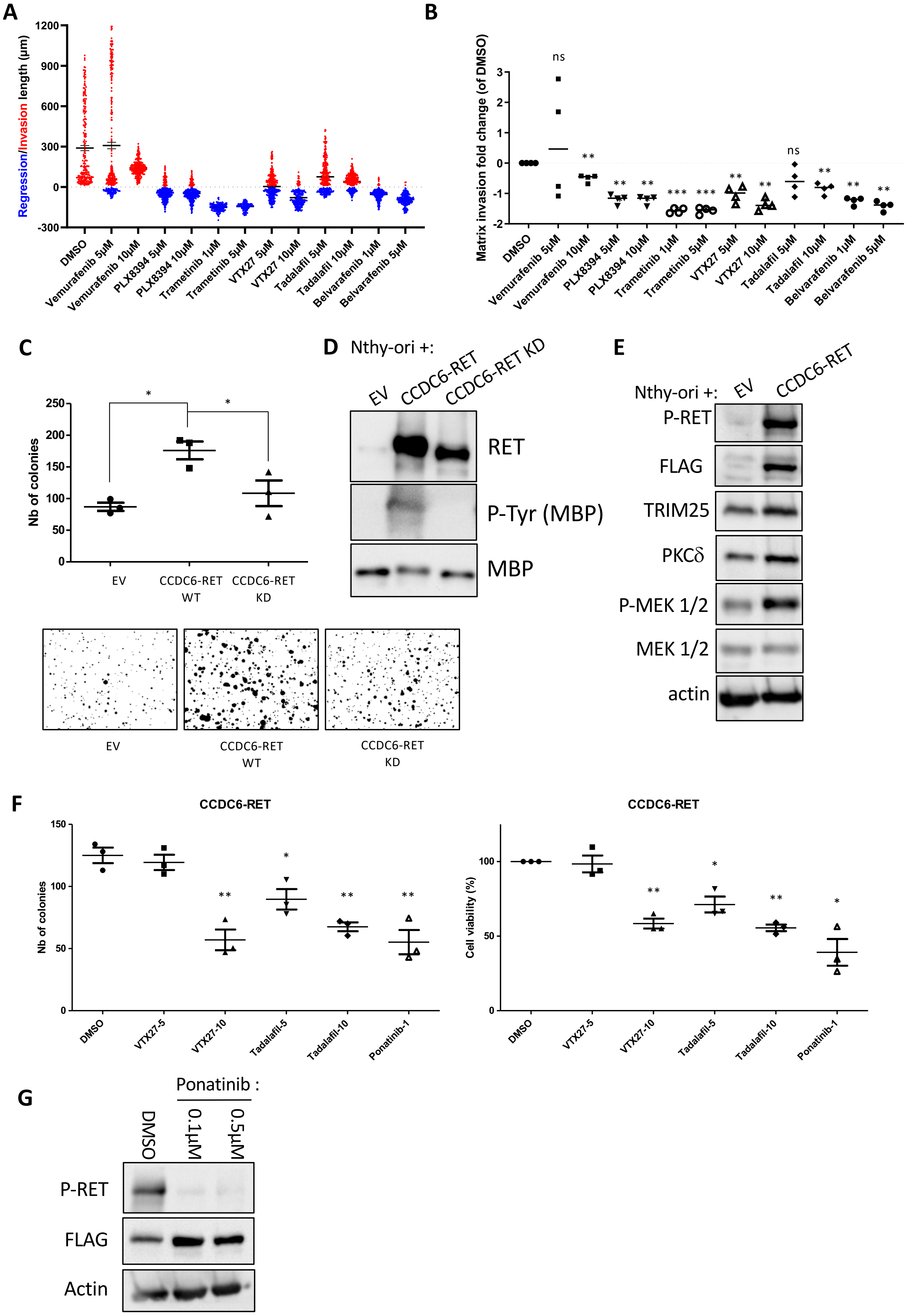

Supplement: Supplementary file 1 — Additional file 1: Suppl. Fig. 1. RET fusions were identified in 3 patients. A. H&E and α-thyroglobulin staining of indicated patients’ tumor tissues. B. Summary of patients’ features. C. Circos plots showing RET fusions detected in patients 14, 15 and 21. D./E. RET fusions detection by qPCR (E) and RT-PCR (F) in indicated samples. F. BAIAP2L1-BRAF detection by qPCR (upper panel) and RT-PCR (lower two panels) in the indicated patients’ samples. The upper graph shows three technical replicates. Suppl. Fig. 2. Numerous thyroid-related genes are dysregulated in tumor tissue compared to normal tissue. A. Heat map of RNA-seq analysis of normal vs tumor tissue of the 4 patients carrying the fusion proteins (adjusted p-value < 0.05, log2 fold change ≠ 0). B. Comparison of RNA-seq data of 19 thyroid-related genes. Patient 17 is here compared to “all patients” representing the three pateints 14, 15 and 21. C. Individual normalized read counts of 2 thyroid-related genes, TPO and DIO1. D. Determination of the relative expression of TPO and DIO1 in tumor and metastasis vs normal samples of all four patients by qPCR. Three technical replicates of each patient are shown (error bars = SEM). E. Determination of the relative expression of TPO and DIO1 in normal patient 17 vs other patients’ normal samples by qPCR (error bars = SEM, three technical replicates). Suppl. Fig. 3. TRIM25 and PKCδ are not upregulated at the mRNA level. A. Heat map of mass spectrometry analysis of normal vs tumor vs metastasis. B. Relative protein intensities from mass spectrometric data of PKCδ, TRIM25 and IQGAP1 from the individual patients. C. Differential expression (log2(fold change)) of PKCδ, TRIM25 and IQGAP1 from mass spectrometry data in patients #14, #15, #17 and #21. Each dot represents the log2(fold change) of one patient. D. RNA-seq data showing PKCδ, TRIM25, and IQGAP1 expression in indicated samples. Suppl. Fig. 4. Combination of mass spectrometric vs RNA-seq data allowed identification of ad [file 12943_2022_1665_MOESM1_ESM.pdf]
